# Supplementary material for: Gene therapy prevents hepatic mitochondrial dysfunction in murine deoxyguanosine kinase deficiency
Source: Mol Ther Methods Clin Dev. 2024 Dec 13;33(1):101397. doi: 10.1016/j.omtm.2024.101397 (PMC11782815; doi:10.1016/j.omtm.2024.101397)
Supplement: Document S1. Figures S1–S5 and Table S1 [file mmc1.pdf]

OMTM, Volume *33*

Supplemental information

Gene therapy prevents hepatic mitochondrial dysfunction in murine deoxyguanosine kinase deficiency

Nandaki Keshavan, Miriam Greenwood, Helen Prunty, Juan Antinao Diaz, Riccardo Privolizzi, John Counsell, Anna Karlsson, Neil Sebire, Simon Waddington, Rajvinder Karda, and Shamima Rahman

Figure S1

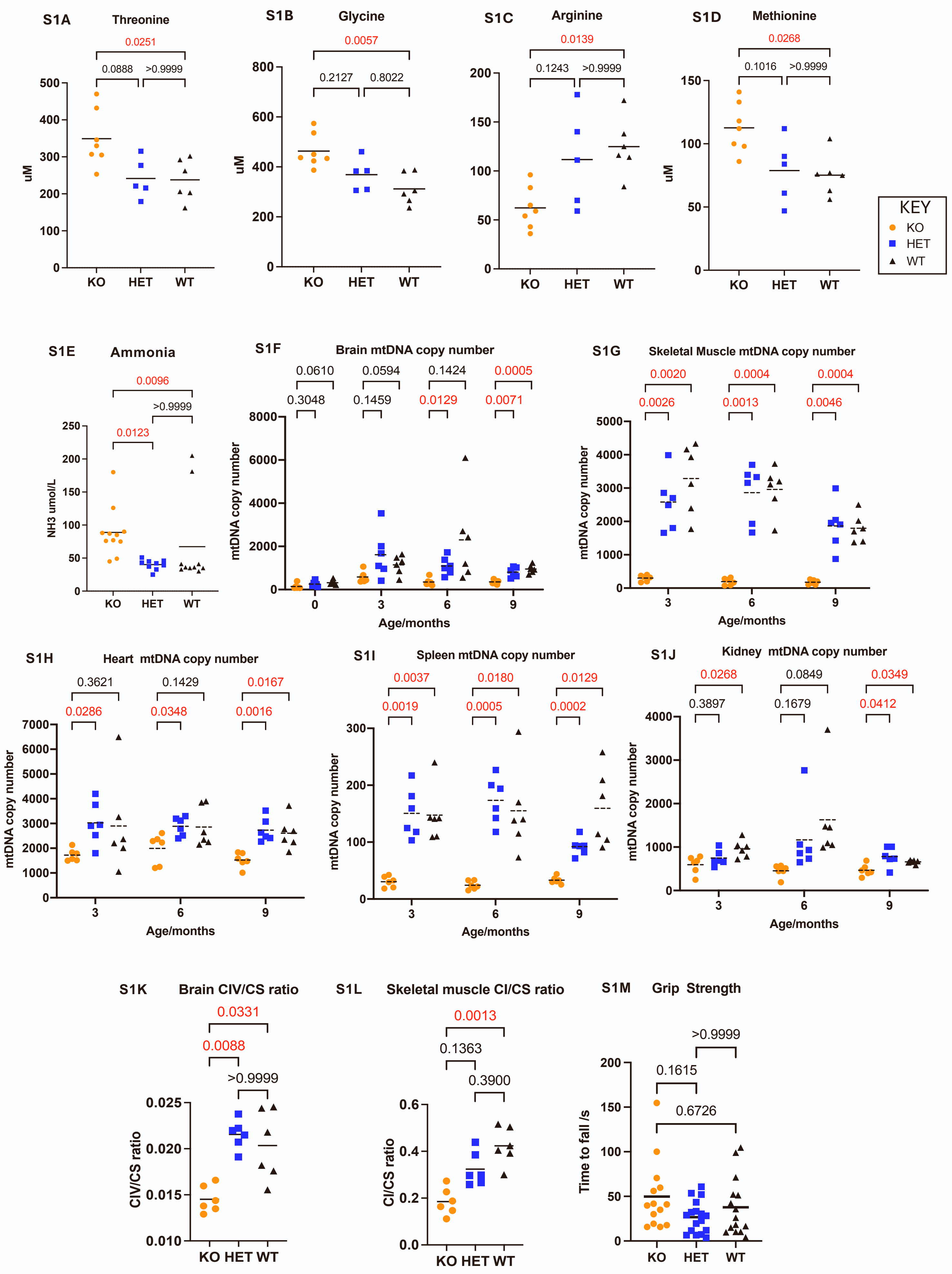
S1A Threonine
0.0251
0.0888 >0.9999
uM
KO HET WT
S1B Glycine
0.0057
0.2127 0.8022
uM
KO HET WT
S1C Arginine
0.0139
0.1243 >0.9999
KO HET WT
S1D Methionine
0.0268
0.1016 >0.9999
uM
KO HET WT
KEY
KO
HET
WT
S1E Ammonia
0.0096
>0.9999
0.0123
NH3 umol/L
KO HET WT
S1F Brain mtDNA copy number
0.0610 0.0594 0.1424 0.0005
0.3048 0.1459 0.0129 0.0071
mtDNA copy number
Age/months
S1G Skeletal Muscle mtDNA copy number
0.0020 0.0004 0.0004
0.0026 0.0013 0.0046
mtDNA copy number
Age/months
S1H Heart mtDNA copy number
0.3621 0.1429 0.0167
0.0286 0.0348 0.0016
mtDNA copy number
Age/months
S1I Spleen mtDNA copy number
0.0037 0.0180 0.0129
0.0019 0.0005 0.0002
mtDNA copy number
Age/months
S1J Kidney mtDNA copy number
0.0268 0.0849 0.0349
0.3897 0.1679 0.0412
mtDNA copy number
Age/months
S1K Brain CIV/CS ratio
0.0331
0.0088
>0.9999
CIV/CS ratio
KO HET WT
S1L Skeletal muscle CI/CS ratio
0.0013
0.1363
0.3900
CI/CS ratio
KO HET WT
S1M Grip Strength
>0.9999
0.1615
0.6726
Time to fall /s
KO HET WT

Figure S1 Additional baseline phenotyping data for Dguok KO mouse model

S1A-S1D: Serum amino acid measurements for threonine, glycine, arginine and methionine. Threonine, glycine and methionine were significantly increased whereas arginine was reduced in KO mice compared to WTs. Sample sizes: 7KO, 5HET, 6WT. Statistics: Kruskal-Wallis test for multiple comparisons. **S1E:** Blood ammonia measurements at 9 months. Hyperammonaemia was demonstrated in KOs compared to HET and WT mice. Sample sizes: 11KO, 9HET, 10WT. Statistics: Kruskal-Wallis test for multiple comparisons. **S1F:** Brain mtDNA quantitation. KOs showed significant brain mtDNA depletion compared to WTs at 9 months but not prior to this time point. Sample size: 6 per genotype per time point. Statistics: 2-way ANOVA with Tukey's test for multiple comparisons. **S1G:** Skeletal muscle mtDNA quantitation. Skeletal muscle mtDNA depletion was demonstrated from 3 months onward. Sample size: 6 per genotype per time point. Statistics: 2-way ANOVA with Tukey's test for multiple comparisons. **S1H** Heart mtDNA quantitation. Heart mtDNA deficiency was demonstrated at 9 months. Sample size: 6 per genotype per time point. Statistics: 2-way ANOVA with Tukey's test for multiple comparisons. **S1I:** Spleen mtDNA quantitation. Spleen mtDNA depletion was demonstrated from 3 months onward. Sample size: 6 per genotype per time point. Statistics: 2-way ANOVA with Tukey's test for multiple comparisons. **S1J** Kidney mtDNA quantitation. Kidney mtDNA deficiency was demonstrated at 9 months. Sample size: 6 per genotype per time point. Statistics: 2-way ANOVA with Tukey's test for multiple comparisons. **S1K:** Brain complex IV/CS ratio at 9 months. KOs showed significant brain complex IV deficiency. Sample size: 6 per genotype. Statistics: Kruskal-Wallis test for multiple comparisons. **S1L:** Skeletal muscle complex I/CS ratio at 9 months. KOs showed skeletal muscle complex I deficiency. Sample size: 6 per genotype. Statistics: Kruskal-Wallis test for multiple comparisons. **S1M:** Grip strength testing undertaken as part of behavioural testing. No significant differences were seen between KOs and WT/HETs. Sample sizes: 14KO, 17HET, 15WT. Statistics: Kruskal-Wallis test for multiple comparisons. p values are indicated above the brackets. p values <0.05 are indicated in red.

Figure S2

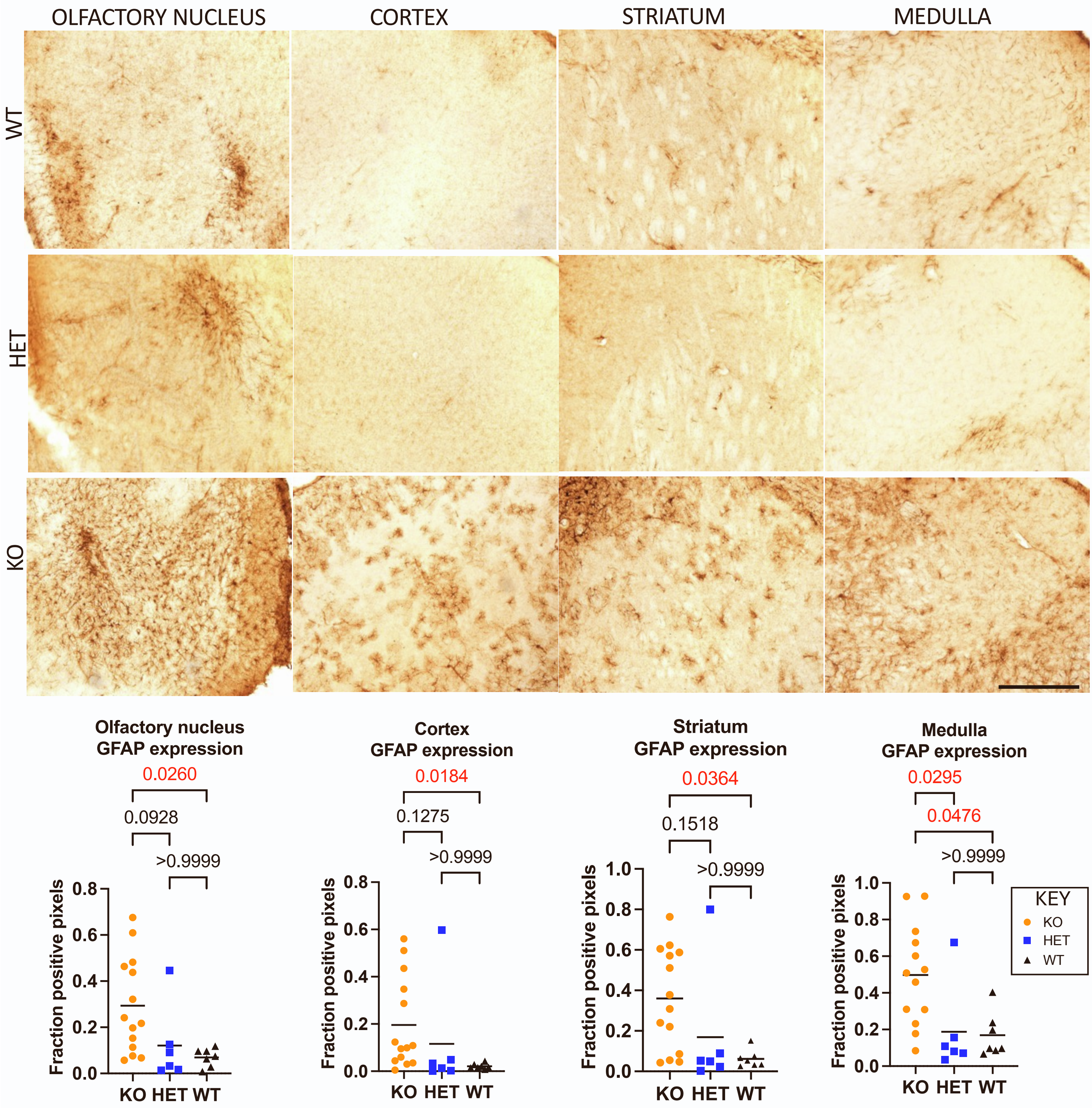

Figure S2 Characterisation of neurological phenotype in murine *Dguok* KO model. Neuroinflammatory phenotyping demonstrated widespread neuroinflammation in *Dguok* KO mice at 9 months. Astrogliosis was evident on anti-GFAP immunohistochemistry in diffuse brain regions (olfactory nucleus, cortex, striatum and medulla oblongata) of KO mice. Images displayed are representative of the mean expression for each genotype and were taken at x10 magnification, scale bar: 500µm. Graphs show quantitation of staining as determined by thresholding. Sample size: 7 WT, 6 HET, 14 KOs for olfactory nucleus, cortex, striatum and 7 WT, 6 HET, 13 KOs for medulla. Statistics: Kruskal Wallis test with multiple comparisons. p values <0.05 are highlighted red.

Figure S3

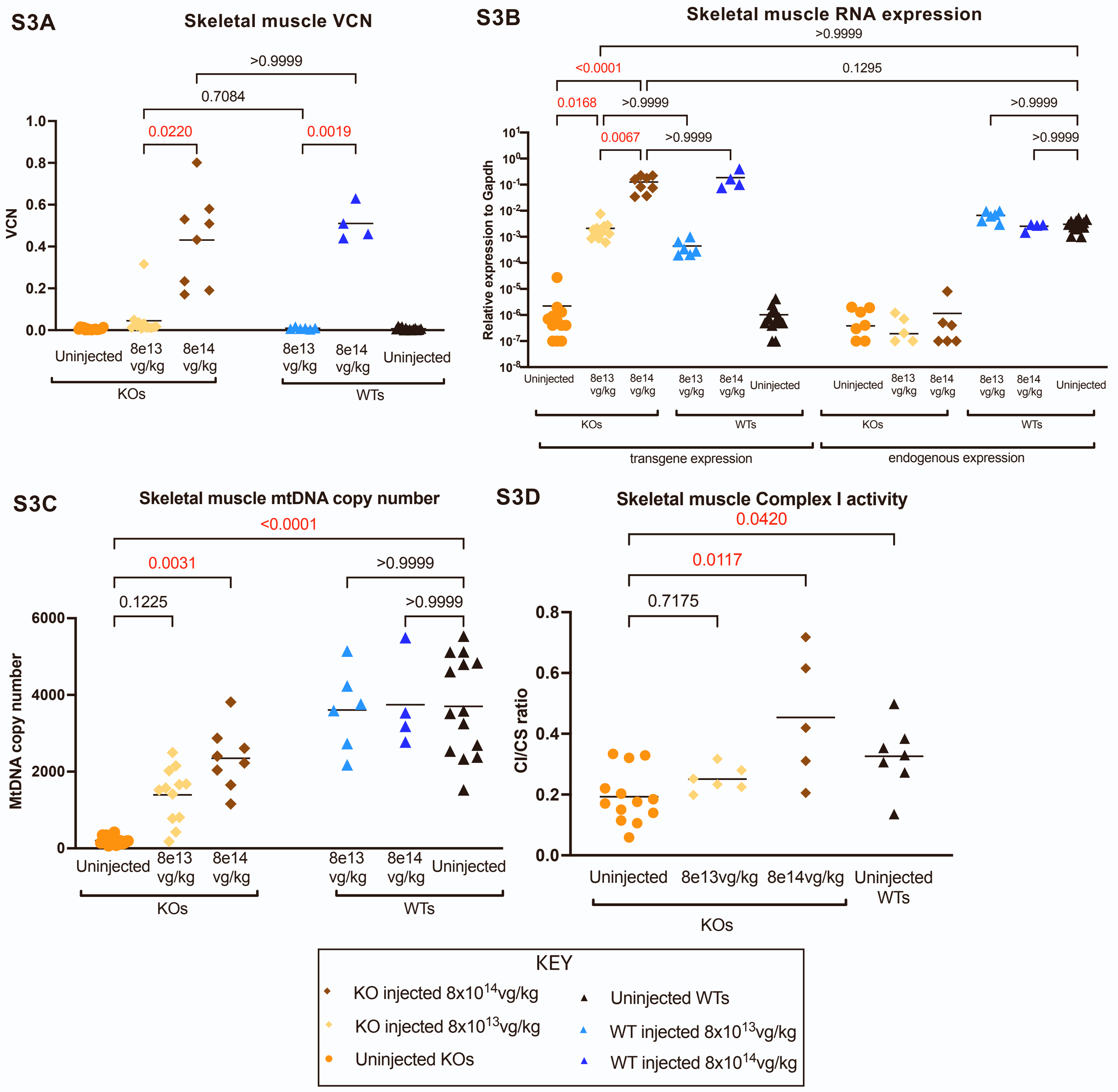

Figure S3 Outcomes of neonatal gene transfer in skeletal muscle.
S3A Skeletal muscle VCN data in injected KO and WT mice. VCN data show low but dose-dependent cell transduction in skeletal muscle following IV gene transfer. **S3B** Skeletal muscle RNA expression data. Skeletal muscle transgene (*hDGUOK)* expression in KOs injected at 8e13vg/kg and 8e14vg/kg is not statistically different to endogenous WT *mDguok* RNA expression**. S3C** Skeletal muscle mtDNA copy number data in injected KO and WT mice**.** mtDNA copy number is partially rescued following IV gene transfer at a dose of 8e14vg/kg. Sample sizes for VCN, RNA expression and mtDNA copy number studies: 16 uninjected KOs, 12 KOs injected with 8e13vg/kg, 8 KOs injected with 8e14vg/kg, 6 WT injected with 8e13vg/kg, 4 WT injected with 8e14vg/kg, 14 uninjected WTs. Statistics: Kruskal-Wallis test with multiple comparisons. **S3D** Skeletal muscle complex I activity in injected KO mice. Complex I deficiency is rescued in KOs injected at the 8e14vg/kg dose but not the 8e13vg/kg dose. Sample sizes: 13 uninjected KOs, 6 KOs injected with 8e13vg/kg, 5 KOs injected with 8e14vg/kg, 7 uninjected WTs. Statistics: Kruskal-Wallis test with multiple comparisons.

Figure S4

S4A

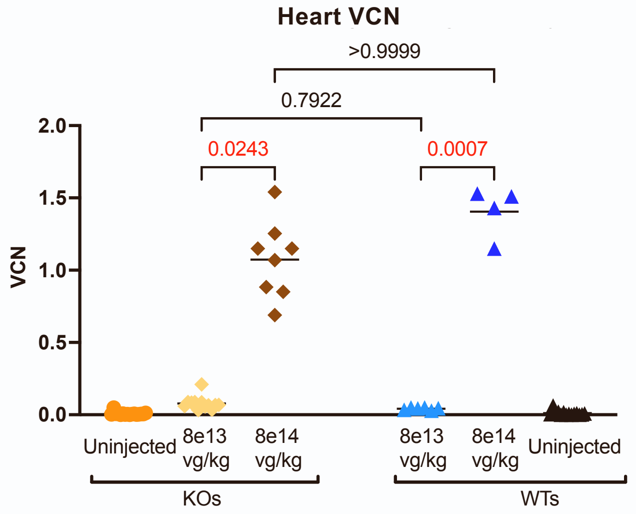

S4B

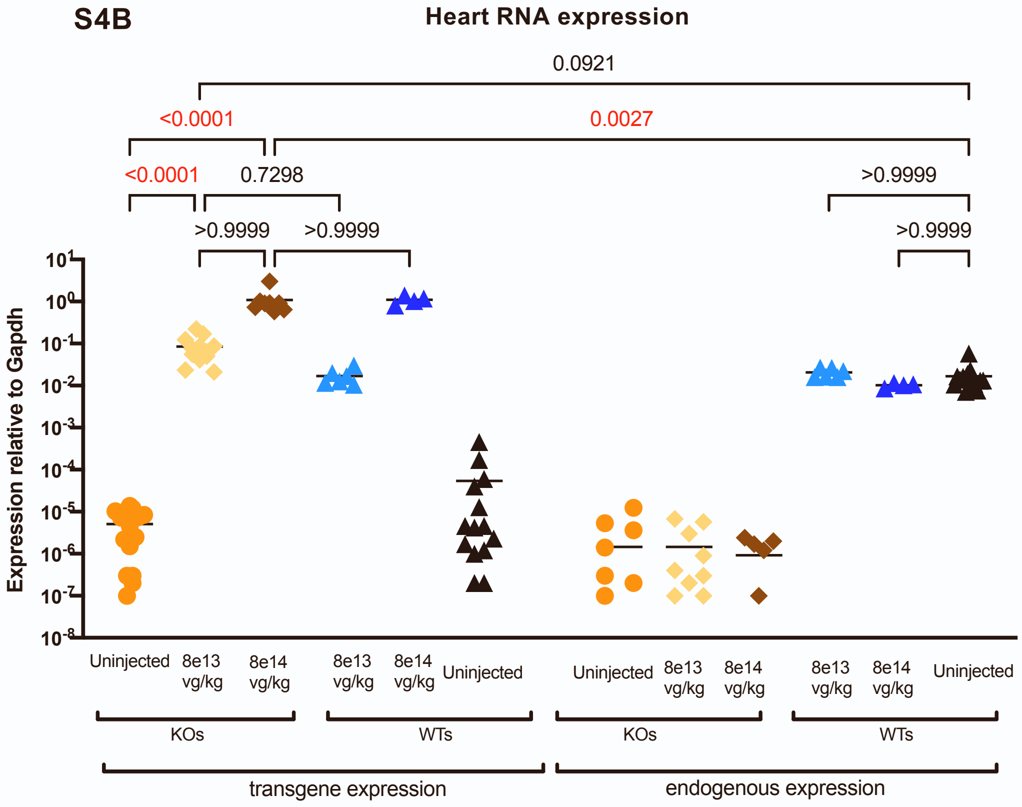

S4C

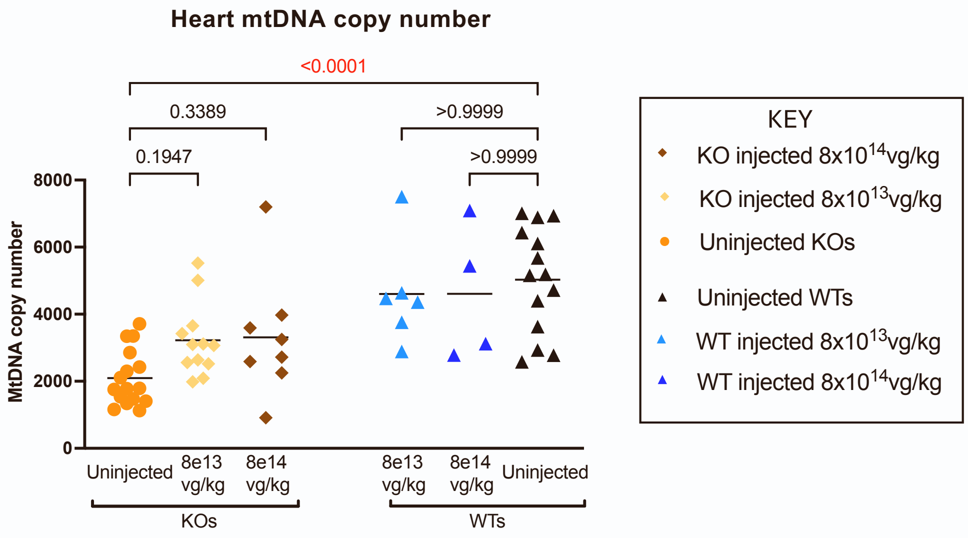

Figure S4 Outcomes of neonatal gene transfer in heart.

S4A Cardiac VCN data in injected KO and WT mice. VCN data show good transduction at doses of 8e14vg/kg following IV gene transfer.

S4B Cardiac RNA expression data in injected KO and WT mice**.** Cardiac transgene (*hDGUOK)* expression in KOs injected at 8e13vg/kg is not statistically different to endogenous WT *mDguok* RNA expression whereas KOs injected at 8e14vg/kg demonstrated higher transgene expression compared to endogenous WT *mDguok* expression**.**

S4C Cardiac mtDNA copy number data in injected KO and WT mice. mtDNA copy number is not significantly rescued following IV gene transfer.

Sample sizes for VCN, RNA expression and mtDNA copy number studies: 16 uninjected KOs, 12 KOs injected with 8e13vg/kg, 8 KOs injected with 8e14vg/kg, 6 WT injected with 8e13vg/kg, 4 WT injected with 8e14vg/kg, 14 uninjected WTs. Statistics: Kruskal-Wallis test with multiple comparisons.

Figure S5

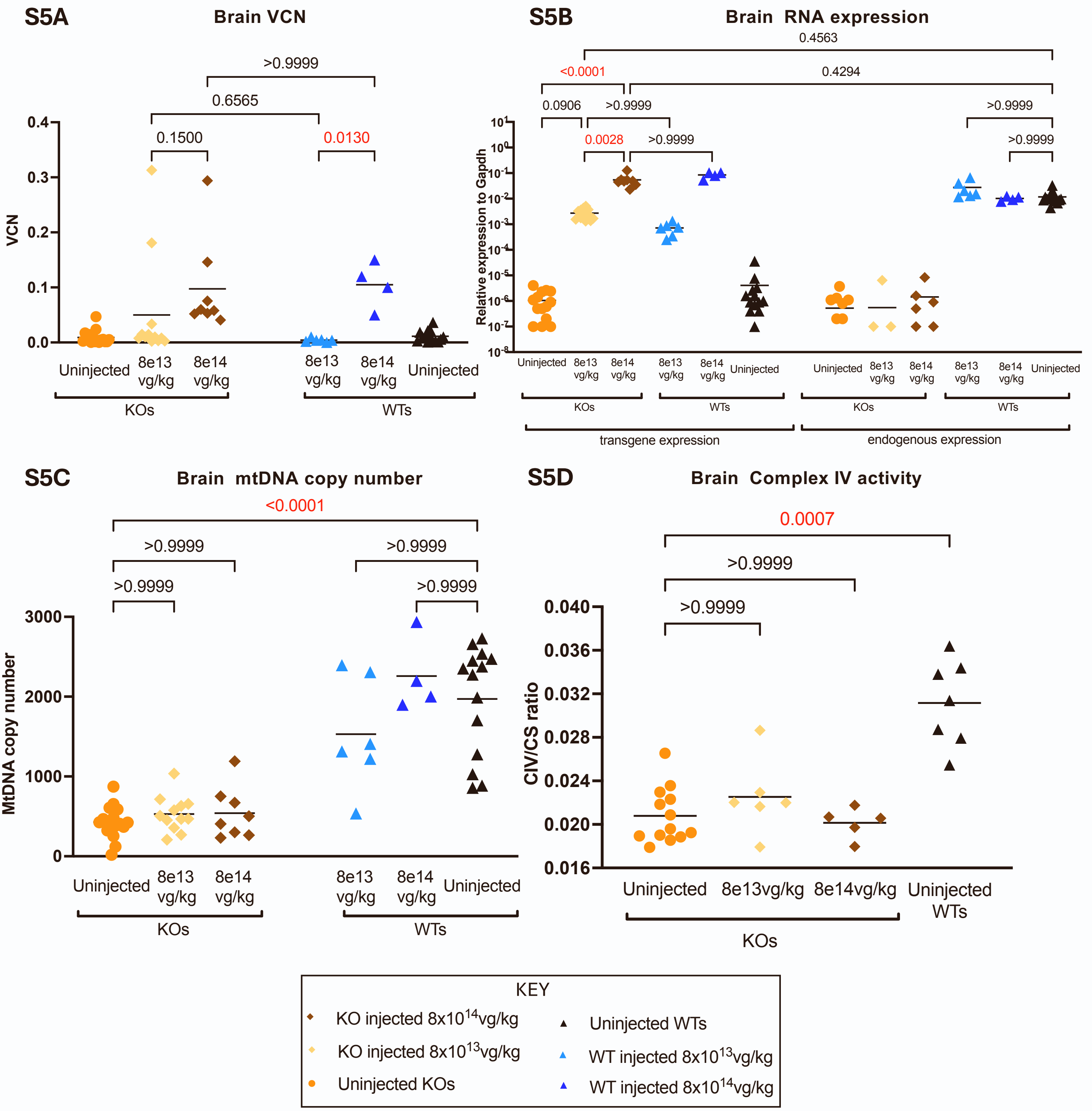

S5A
Brain VCN
VCN
>0.9999
0.6565
0.1500
0.0130
0.4
0.3
0.2
0.1
0.0
Uninjected
8e13 vg/kg
8e14 vg/kg
8e13 vg/kg
8e14 vg/kg
Uninjected
KOs
WTs
S5B
Brain RNA expression
0.4563
<0.0001
0.4294
0.0906
>0.9999
>0.9999
0.0028
>0.9999
>0.9999
Relative expression to Gapdh
Uninjected
8e13 vg/kg
8e14 vg/kg
8e13 vg/kg
8e14 vg/kg
Uninjected
Uninjected
8e13 vg/kg
8e14 vg/kg
8e13 vg/kg
8e14 vg/kg
Uninjected
KOs
WTs
KOs
WTs
transgene expression
endogenous expression
S5C
Brain mtDNA copy number
<0.0001
>0.9999
>0.9999
>0.9999
>0.9999
MtDNA copy number
3000
2000
1000
0
Uninjected
8e13 vg/kg
8e14 vg/kg
8e13 vg/kg
8e14 vg/kg
Uninjected
KOs
WTs
S5D
Brain Complex IV activity
0.0007
>0.9999
>0.9999
CIV/CS ratio
0.040
0.036
0.032
0.028
0.024
0.020
0.016
Uninjected
8e13vg/kg
8e14vg/kg
Uninjected WTs
KOs
KEY
KO injected 8x10^14vg/kg
KO injected 8x10^13vg/kg
Uninjected KOs
Uninjected WTs
WT injected 8x10^13vg/kg
WT injected 8x10^14vg/kg

Figure S5 Brain outcomes following neonatal gene transfer

S5A Brain VCN data in injected KO and WT mice. VCN data show low cell transduction in brain following IV gene transfer. **S5B** Brain RNA expression data. Brain transgene (*hDGUOK)* expression in KOs injected at 8e13vg/kg and 8e14vg/kg is not statistically different to endogenous WT *mDguok* RNA expression**. S5C** Brain mtDNA copy number data in injected KO and WT mice**.** mtDNA copy number is not rescued following IV gene transfer. Sample sizes for VCN, RNA expression and mtDNA copy number studies: 16 uninjected KOs, 12 KOs injected with 8e13vg/kg, 8 KOs injected with 8e14vg/kg, 6 WTs injected with 8e13vg/kg, 4 WTs injected with 8e14vg/kg, 14 uninjected WTs. Statistics: Kruskal-Wallis test with multiple comparisons. **S5D** Brain complex IV activity in injected KO mice. Complex IV deficiency is persistent in injected KO mice despite IV gene transfer. Sample sizes: 13 uninjected KOs, 6 KOs injected with 8e13vg/kg, 5 KOs injected with 8e14vg/kg, 7 uninjected WTs. Statistics: Kruskal-Wallis test with multiple comparisons.

Table S1 OXPHOS measurements in baseline phenotyping

Data are expressed as mean±SD. Significantly decreased activity (indicated in blue) is seen in liver in KOs compared to WTs for complex I (p=0.0006), complex II+III (p=0.038), complex III (p=0.024), complex IV (p=0.002), in brain for complex IV (p=0.03) and in skeletal muscle for complex I (p= 0.0013). Significantly increased citrate synthase activity (indicated in red) is seen in liver of KOs compared to WTs, p=0.008. Sample size: 6 mice per genotype. Statistics performed using one-way ANOVA for multiple comparisons.

Organ		Complex I ratio	Complex II+III ratio	Complex III ratio	Complex IV ratio	Citrate synthase nmol/min/mg
**Liver**	KO	0.348±0.07	0.151±0.05	0.034±0.01	0.023±0.005	62±7
	HET	0.541±0.06	0.475±0.13	0.089±0.03	0.063±0.013	55±13
	WT	0.789±0.17	0.424±0.15	0.084±0.01	0.083±0.027	41±6
**Brain**	KO	0.154±0.03	0.055±0.02	0.033±0.01	0.014±0.001	170±21
	HET	0.218±0.02	0.052±0.03	0.058±0.007	0.021±0.001	180±32
	WT	0.177±0.03	0.048±0.02	0.046±0.01	0.020±0.003	179±61
**Skeletal muscle**	KO	0.185±0.05	0.080±0.04	0.020±0.02	0.014±0.003	74±15
	HET	0.324±0.07	0.144±0.02	0.034±0.02	0.019±0.006	70±24
	WT	0.423±0.08	0.140±0.04	0.045±0.02	0.019±0.004	86±31
**Heart**	KO	0.529±0.10	0.034±0.01	0.009±0.004	0.007±0.001	397±56
	HET	0.436±0.14	0.019±0.01	0.008±0.005	0.005±0.002	463±113
	WT	0.451±0.05	0.027±0.02	0.006±0.002	0.005±0.001	463±53
