# Supplementary material for: Gene therapy prevents hepatic mitochondrial dysfunction in murine deoxyguanosine kinase deficiency
Source: Mol Ther Methods Clin Dev. 2024 Dec 13;33(1):101397. doi: 10.1016/j.omtm.2024.101397 (PMC11782815; doi:10.1016/j.omtm.2024.101397)
Supplement: Document S2. Article plus supplemental information [file mmc2.pdf]

Molecular Therapy
Methods & Clinical Development
Original Article

Gene therapy prevents hepatic mitochondrial dysfunction in murine deoxyguanosine kinase deficiency

Nandaki Keshavan,[1,2,3] Miriam Greenwood,[1] Helen Prunty,[4] Juan Antinao Diaz,[3] Riccardo Privolizzi,[3] John Counsell,[1] Anna Karlsson,[5] Neil Sebire,[6] Simon Waddington,[3] Rajvinder Karda,[3] and Shamima Rahman[1,2]

[1]UCL GOS Institute of Child Health, 30 Guilford Street, London WC1N 1EH, UK; [2]Department of Metabolic Medicine, Great Ormond Street Hospital NHS Trust, Guilford St, London WC1N 3BH, UK; [3]UCL EGA Institute for Women's Health, 86-96 Chenies Mews, London WC1E 6HX, UK; [4]Department of Chemical Pathology, Great Ormond Street Hospital NHS Trust, Guilford St, London WC1N 3BH, UK; [5]Division of Clinical Microbiology, Department of Laboratory Medicine, Karolinska Institute, Karolinska University Hospital, 141 86 Stockholm, Sweden; [6]NIHR GOSH Biomedical Research Centre, 30 Guilford Street, London WC1N 1EH, UK

Primary mitochondrial disorders are a cause of neonatal liver failure. Biallelic pathogenic variants of the gene encoding the mitochondrial localizing enzyme deoxyguanosine kinase (DGUOK) cause hepatocerebral mitochondrial DNA depletion syndrome, leading to acute neonatal liver failure and early mortality. There are currently no effective disease-modifying therapies. In this study, we developed an adeno-associated virus 9 (AAV9) gene therapy approach to treat a mouse model of DGUOK deficiency that recapitulates human disease. We delivered AAV9-*hDGUOK* intravenously to newborn *Dguok* knock-out mice and showed that liver dysfunction was prevented in a dose-dependent manner. Unexpectedly for neonatal delivery, durable and long-lasting liver transduction and RNA expression were observed. Liver mitochondrial DNA depletion, deficiencies of oxidative phosphorylation complexes I, III, and IV and liver transaminitis and survival were ameliorated in a dose-dependent manner.

INTRODUCTION

Mitochondrial DNA (mtDNA) depletion syndromes (MDDS) comprise a heterogeneous subgroup of severe primary mitochondrial disorders caused by mutations in nuclear genes encoding key proteins involved in mtDNA replication or maintenance of mitochondrial deoxyribonucleotide triphosphate pools.[1] Nucleotides incorporated into mtDNA are either synthesized *de novo* or salvaged from the cytosol via a series of biochemical steps known as the mitochondrial salvage pathway. Defects in any of approximately 14 genes result in mtDNA depletion and therefore loss of the mtDNA-encoded subunits of oxidative phosphorylation (OXPHOS) enzymes, and lead to severe energy deficiency frequently presenting as a debilitating infantile-onset disease with a high mortality.[2]

Biallelic pathogenic variants in *DGUOK* encoding deoxyguanosine kinase (DGUOK), a component of the mitochondrial nucleoside salvage pathway, account for up to 20% of MDDS.[3] DGUOK catalyzes the intramitochondrial phosphorylation of dG and dA to dGMP and dAMP, respectively.[4] Loss of DGUOK function results in imbalanced nucleotide pools, nucleotide misincorporation, and mtDNA depletion.[5] Neonates and infants with DGUOK deficiency typically present with severe acute liver failure which correlates with early mortality. Current management is only supportive. Advances in adeno-associated virus (AAV)-based gene therapy technology and subsequent clinical trial successes have led to market approval for some genetic disorders, but not yet for primary mitochondrial diseases.

Here we used a mouse model of DGUOK deficiency, which closely recapitulates the human disease phenotype.[6] The model, generated by disruption of exon 2 of *Dguok* using Cre/lox homologous recombination, demonstrates weight loss and chronic liver disease. In this study, we aimed to develop an AAV9-based gene therapy approach to prevent liver mitochondrial dysfunction in this model. We showed that AAV9 mediates efficient lasting liver transduction, enabling rescue of mtDNA depletion, OXPHOS abnormalities, and transaminitis, and a dose-dependent improvement in survival in knock-out (KO) mice.

RESULTS

Dguok KO mice recapitulate the human disease phenotype

KO mice exhibited liver mtDNA depletion from birth with mtDNA levels of approximately 29% of wild-type (WT) and heterozygous (HET) controls. Liver mtDNA copy number decreased further to less than 5% of WT/HET controls by 3 months of age and remained at this level thereafter (Figure 1A).

Elevations of blood alanine aminotransferase (ALT), aspartate aminotransferase (AST), and alkaline phosphatase ALP) (Figures 1B–1D), amino acids threonine, glycine, arginine, and methionine (Figures S1A–S1D), and ammonia (Figure S1E) were seen in KO mice. OXPHOS

Received 2 June 2024; accepted 10 December 2024;
https://doi.org/10.1016/j.omtm.2024.101397.

Correspondence: Shamima Rahman, UCL GOS Institute of Child Health, 30 Guilford St, London WC1N 1EH, UK
E-mail: shamima.rahman@ucl.ac.uk

Published by Elsevier Inc. on behalf of The American Society of Gene and Cell Therapy.
This is an open access article under the CC BY license (http://creativecommons.org/licenses/by/4.0/).

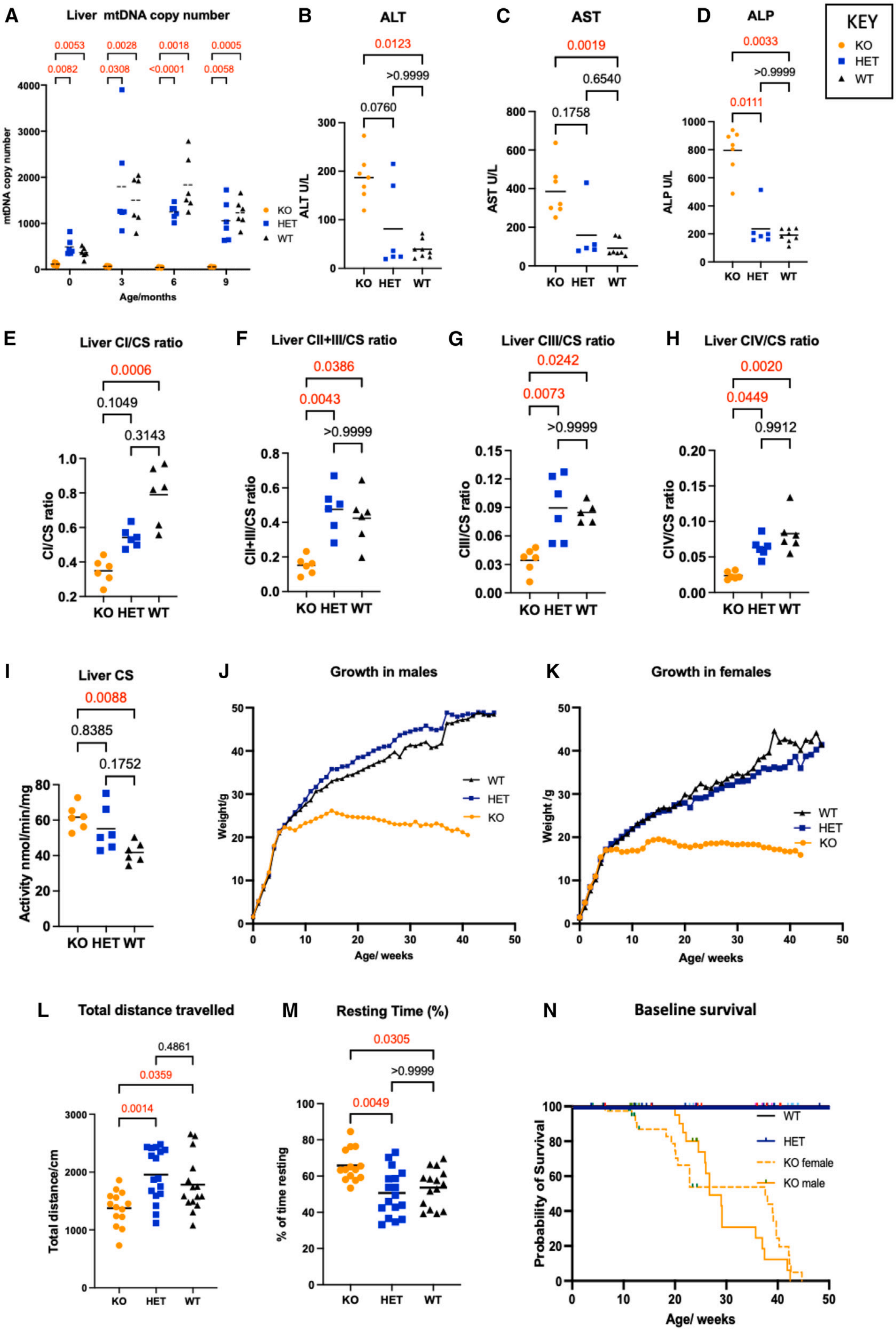

(legend on next page)

activities in tissue homogenates from mice at 9 months of age revealed multiple OXPHOS deficiencies in liver of KO mice compared with WTs. Complex I, II + III, III, and IV activities were deficient, together with significant elevation of citrate synthase activity, reflecting a compensatory increase in mitochondrial mass (Table S1; Figures 1E–1I).

The mean mtDNA copy numbers in the brain were approximately 50% of WT/HET levels at birth, although this difference was not statistically significant (KO 142, HET 253, WT 319; KO vs. HET: p = 0.30; KO vs. WT: p = 0.06). However, at 9 months mtDNA copy number was significantly decreased to approximately 40% of WT/HETs (KO 356, HET 805, WT: 952; KO vs. HET: p = 0.007; KO vs. WT: p = 0.0005 (Figure S1F). From a biochemical perspective, the brain was affected more mildly, showing only isolated complex IV deficiency (Figure S1K). Increased glial fibrillary acidic protein (GFAP) expression was observed throughout the brain of some KO mice, but this finding was variable. Statistically significant increases in GFAP expression in KOs compared with WTs were observed in olfactory nucleus, cortex, striatum, and medulla oblongata (Figure S2).

MtDNA depletion was also observed in skeletal muscle, heart, and spleen and a decrease of mtDNA copy number was seen in kidney (Figures S1G–S1J). Complex I deficiency was seen in skeletal muscle (Figure S1L). No OXPHOS abnormalities were observed in heart (Table S1).

Growth velocity of KOs decreased from 6 weeks of age in both sexes. KOs reached a maximum weight (19 g in females and 25 g in males) at approximately 16 weeks, after which their weights plateaued and eventually declined (Figures 1J and 1K). On open field testing, KOs demonstrated significantly lower total distance traveled and increased resting time, as shown in Figures 1L and 1M. There was no significant difference in grip strength in KO mice (Figure S1M).

The humane endpoint was defined as 15% weight loss from the highest measured weight. All KO mice reached this endpoint by 42 weeks, whereas all WT and HET mice survived (Figure 1N). Female and male KOs had median survival of 37.5 and 26.7 weeks, respectively. This difference was statistically significant (Mantel-Cox test, $p < 0.0001$).

Gene transfer ameliorates liver disease in *Dguok* KO mice

Of all organs, the highest transduction was observed in liver. Vector copy number (VCN) was significantly higher in the 8×10^{14} vg/kg KO group (mean VCN 5.03) than the 8×10^{13} vg/kg KO group (mean VCN 1.57, p = 0.0116), indicating a dose response (Figure 2A). VCN data in skeletal muscle and heart showed lower transduction than liver (Figures S3A and S4A). In liver, *hDGUOK* expression normalized to *mGapdh* in injected KOs exceeded endogenous *mDguok* expression at both doses (8×10^{13} vg/kg KOs: mean *hDGUOK* expression 1.72, 8×10^{14} vg/kg KOs: mean *hDGUOK* expression 1.81, uninjected WT *mDguok* expression: 0.018, corresponding with fold changes of 95 and 100, respectively). There was also greater *hDGUOK* RNA expression in injected KOs compared with dose-matched injected WTs. WT-injected mice had *mDguok* levels that were similar to control uninjected WTs, i.e., no downregulation of endogenous gene expression (Figure 2B).

In biodistribution studies, quantification of GFP expression by immunohistochemistry revealed a greater than 6-fold increase in long-term GFP expression in injected KO liver compared with injected WTs (p = 0.0159). The pattern of GFP positivity was non-uniform, with islands of positive cell clusters interspersed between clusters of negative cells (Figures 2C and 2D).

In skeletal muscle and cardiac tissue, RNA expression showed dose-responsiveness which was supranormal at the 8×10^{14} vg/kg dose (Figures S3B and S4B).

Figure 1. Phenotyping of murine *Dguok* KO model

(A) Liver mtDNA quantitation. KOs showed significant liver mtDNA depletion compared with WT and HET littermates from birth. This was persistent to 9 months. Mean liver mtDNA levels were 30% of WT levels at birth, 4.4% at 3 months, 2.2% at 6 months, and 4.5% at 9 months. Sample sizes: 6 per genotype per time point. Statistics: ANOVA with Tukey's multiple comparisons test. (B–D) Liver function tests taken at 9 months. KOs showed significantly increased blood ALT, AST, and ALP levels compared with WT littermates. ALT: mean for KOs 187, mean for HETs 81, mean for WTs 39 U/L. AST: mean for KOs 385, mean for HETs 159, mean for WTs 91 U/L. ALP: mean for KOs 794, mean for HETs 236, mean for WTs 191 U/L. Sample sizes: ALT and ALP: 7 KOs, 6 HETs, and 8 WTs. AST: 7 KOs, 5 HETs, and 7 WTs. Statistics: Kruskal-Wallis test with multiple comparisons. (E–I) OXPHOS measurements taken at 9 months. Multiple OXPHOS abnormalities demonstrated in liver of KO mice, namely, complex I deficiency (44% of WT levels), complex II + III deficiency (36% of WT levels), complex III deficiency (40% of WT levels), complex IV deficiency (28% of WT levels), and upregulated citrate synthase activity (151% of WT levels). Mitochondrial respiratory chain complex activities are expressed as a ratio to citrate synthase activity. Sample sizes: six per genotype. Statistics: Kruskal-Wallis test with multiple comparisons. (J and K) Weight of mice in long-term follow-up. Female and male KOs showed a decrease in growth velocity compared with WTs from 6 weeks, which reached statistical significance at 9 weeks (Tukey's test with multiple comparisons as part of mixed-effects analysis). In KOs, the highest mean weight was seen at 16 weeks (19 g in females and 25 g in males), after which there was a gradual decline over several months until the humane endpoint. WT and HET mice continued to gain weight progressively in follow-up. Sample size: females, 33 KOs, 18 HETs, and 8 WTs; males, 27 KOs, 8 HETs, and 15 WTs. (L and M) Open field testing at 9 months. KOs show significantly reduced total distance traveled and increased resting time compared with WTs. Mean total distance traveled: KOs 1,376 cm, HETs 1,954 cm, and WTs 1,781 cm. Mean % resting times: KOs 65%, HETs 50%, and WTs 53%. Sample sizes: 14 KOs, 17 HETs, and 17 WTs. Statistics for total distance traveled: One-way ANOVA, Tukey's test with multiple comparisons. Statistics for percent resting time: Kruskal-Wallis test with multiple comparisons. (N) Survival curve of *Dguok* KO mice as determined by reaching the humane endpoint. Upward ticks indicate censored subjects. KO female mice had a median survival of 37.5 weeks and all reached the humane endpoint by 45 weeks. KO males had a median survival of 26.7 weeks and all reached the humane endpoint by 42 weeks. In comparison with WTs Kos, therefore, had significantly decreased survival: p = 0.001 for females and $p < 0.0001$ for males: Sample sizes: 23 WTs, 53 HETs, and 76 KOs. Statistics: Mantel-Cox test. Graphs show means, p values are indicated above the brackets. p values < 0.05 are indicated in red.

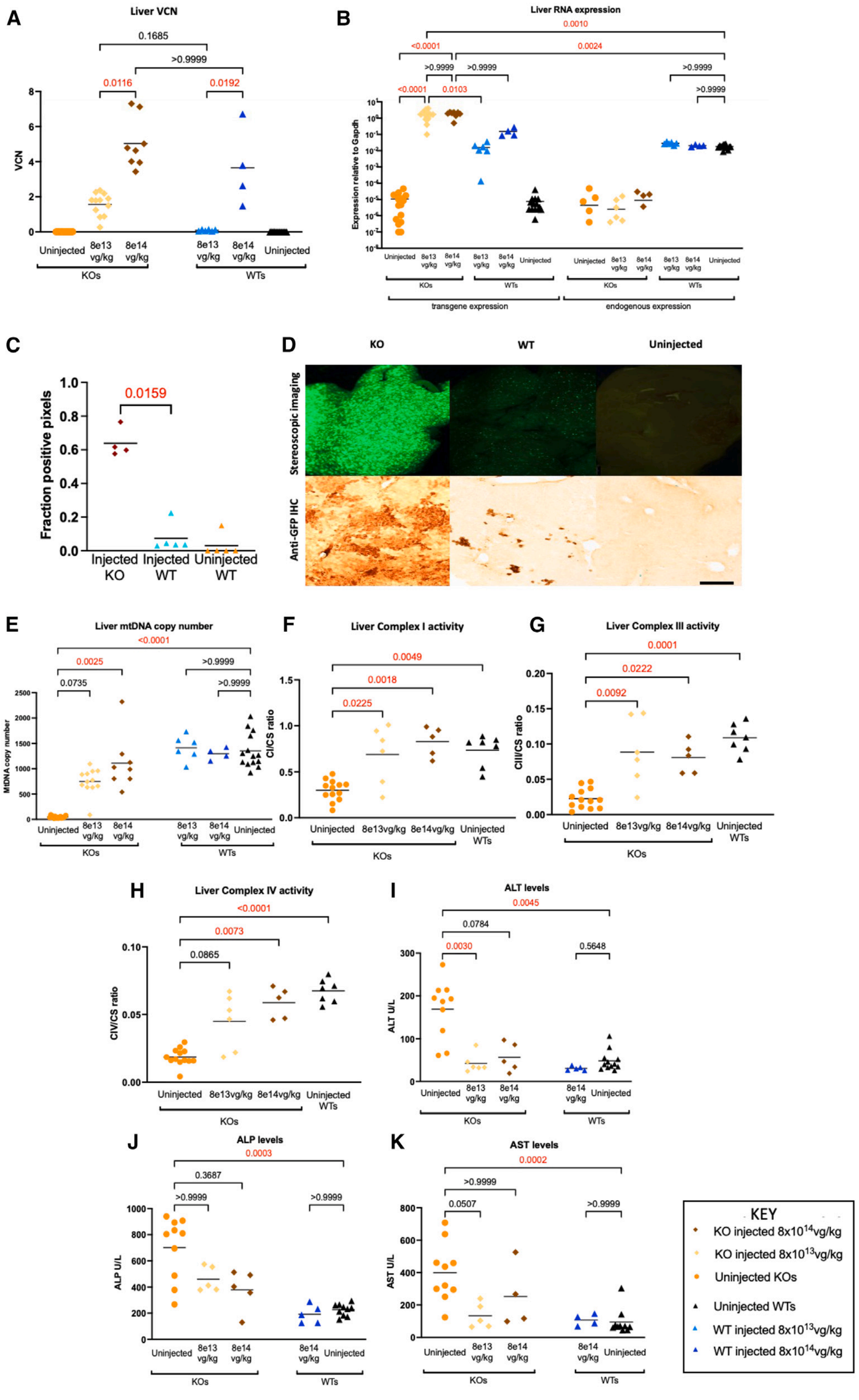

(legend on next page)

Mean liver mtDNA copy number increased from a baseline of 3% in uninjected KOs to 55% in the 8 × 10^{13} vg/kg KO group, but this was not statistically significant (p = 0.07). However, a significant improvement to 82% was observed in the 8 × 10^{14} vg/kg KO group (p = 0.0025) (Figure 2E). No mtDNA abnormality was observed in injected WTs. Dose-dependent improvement in mtDNA copy number was also demonstrated in skeletal muscle (Figure S3C). No significant improvement in heart mtDNA copy number was observed (Figure S4C). Rescue of complex I, III, and IV activities was observed in liver (Figures 2F–2H) and of complex I in skeletal muscle (Figure S3D). Complete rescue of ALT levels was seen at both doses (8 × 10^{13} vg/kg and 8 × 10^{14} vg/kg) in injected KOs, together with partial improvements in AST and ALP. Liver function remained normal in injected WTs (Figures 2I–2K). Histological assessment of livers by hematoxylin and eosin staining showed hepatocellular carcinoma in one KO animal injected with AAV gene therapy at the higher 8 × 10^{14} vg/kg dose but not at the 8 × 10^{13} vg/kg dose (n = 6 per group; data not shown) No hepatocellular carcinoma was seen in uninjected KO or WT mice (n = 7 per group; data not shown).

Gene transfer does not improve brain abnormalities in *Dguok* KO mice

In contrast with the liver, in the brain transduction was poor. VCNs in injected KOs were very low (8 × 10^{14} vg/kg group mean VCN, 0.09; 810^{13} vg/kg group mean VCN, 0.05). No significant difference was observed in mean VCNs between injected KOs and injected WTs (8 × 10^{14} vg/kg group mean VCN: 0.11, 8 × 10^{13} vg/kg group mean VCN: 0.004) (Figure S5A). In brain, injected KOs also showed comparably lower *hDGUOK* RNA expression than in liver, and *hDGUOK* expression was not significantly different from WT endogenous *mDguok* expression at either dose (Figure S5B). In biodistribution studies, GFP expression, although present, was generally low (data not shown). There was persistent mtDNA depletion (22% of WT levels in uninjected KOs, 26% of WT levels in the 8 × 10^{13} vg/kg KO group and 27% of WT levels in the 8 × 10^{14} vg/kg KO group) (Figure S5C) associated with persistent complex IV deficiency at both doses (Figure S5D).

Growth, locomotor, and survival outcomes

We observed minimal improvement of growth in KO injected female mice and no improvement in males after gene therapy. Growth was

Figure 2. Efficacy of liver-directed gene therapy

(A) Liver VCN data after IV gene therapy. VCN data show excellent transduction in liver in a dose-dependent manner. Mean VCNs: 8 × 10^{13} vg/kg injected KOs 1.57, 8 × 10^{14} vg/kg injected KOs 5.0, 8 × 10^{13} vg/kg injected WTs 0.09, and 8 × 10^{14} vg/kg, injected WTs 3.65. Sample sizes: 16 uninjected KOs, 12 KOs injected with 8 × 10^{13} vg/kg, 8 KOs injected with 8 × 10^{14} vg/kg, 6 WTs injected with 8 × 10^{13} vg/kg, 4 WT injected with 8 × 10^{14} vg/kg, and 14 uninjected WTs. Statistics: Kruskal-Wallis test with multiple comparisons. (B) Liver RNA expression. The vertical axis represents target gene expression calculated as a ratio to *mGapdh* and is logarithmic. Liver transgene (*hDGUOK)* expression in KOs injected at 8 × 10^{13} vg/kg and 8 × 10^{14} vg/kg were not significantly different from each other (means 1.72 and 1.81, respectively). However, both were significantly higher than endogenous WT *mDguok* RNA expression (mean 0.018), implying very high transgene expression. Statistics: Kruskal-Wallis test with multiple comparisons. (C) GFP expression in biodistribution studies of mice injected with IV AAV9-*hDGUOK*-GFP. GFP quantitation was performed on images acquired at ×40 magnification after applying thresholding analysis. The data demonstrate significantly higher GFP expression in livers of injected KO mice compared with injected WT mice. Statistical analysis: Mann-Whitney U test comparing the two injected groups only, p value shown above the bracket. Scale bar, 500 μm. Sample sizes: injected KOs: 4, injected WTs: 5, uninjected WTs: 5. (D) Stereoscopic imaging, anti-GFP immunohistochemistry in liver in long-term biodistribution study. Stereoscopic imaging (top): Exposure was identical for the three groups (700 ms). Immunohistochemical images (bottom): images were acquired at ×10 magnification. In both cases, representative images are used. Both show higher GFP expression in injected KO mouse livers compared with injected WT mice. (E) Liver mtDNA copy number data in gene therapy study. mtDNA depletion is significantly rescued in the 8 × 10^{14} vg/kg group, whereas in all but one mouse injected at 8 × 10^{13} vg/kg there is an amelioration of mtDNA depletion. No effect on mtDNA copy number is seen in injected WTs. Mean mtDNA for each group: uninjected KOs 47, KOs injected with 8 × 10^{13} vg/kg 750, KOs injected with 8 × 10^{14} vg/kg 1,112, WTs injected with 8 × 10^{13} vg/kg 1,414, WTs injected with 8 × 10^{14} vg/kg 1,297, uninjected WTs 1,353. Sample sizes: 16 uninjected KOs, 12 KOs injected with 8 × 10^{13} vg/kg, 8 KOs injected with 8 × 10^{14} vg/kg, 6 WTs injected with 8 × 10^{13} vg/kg, 4 WT injected with 8 × 10^{14} vg/kg, and 14 uninjected WTs. Statistics: Kruskal-Wallis test with multiple comparisons. (F–H) Liver complex I, III, and IV activity after gene therapy. The data show an improvement of complex I activity from a baseline of 41% of WT levels in uninjected KO mice to 93% of WT levels in the 8 × 10^{13} vg/kg injected KO group and 112% of WT levels in the 8 × 10^{14} vg/kg injected KO group. At both doses, this improvement was statistically significant compared with uninjected KOs. Similarly, complex III deficiency was ameliorated from a baseline of 21% of WT levels in uninjected KO mice to 81% of WT levels in the 8 × 10^{13} vg/kg injected KO group and 73% of WT levels in the 8 × 10^{14} vg/kg injected KO group. At both doses, this improvement was statistically significant compared with uninjected KOs. Complex IV deficiency was ameliorated from a baseline of 28% of WT levels in uninjected KO mice to 67% of WT levels in the 8 × 10^{13} vg/kg injected KO group and 87% of WT levels in the 8 × 10^{14} vg/kg injected KO group. This was significant only for 8 × 10^{14} vg/kg group, but not the 8 × 10^{13} vg/kg group. Mitochondrial respiratory chain complex activities are expressed as a ratio to citrate synthase activity. Sample sizes: 13 uninjected KOs, 6 KOs injected with 8 × 10^{13} vg/kg, 5 KOs injected with 8 × 10^{14} vg/kg, and 7 uninjected WTs. Statistics: Kruskal-Wallis test with multiple comparisons. (I–K) Liver function tests after gene therapy. There was an improvement in ALT from a baseline mean of 169 U/L in uninjected KOs to 42 U/L in the 8 × 10^{13} vg/kg KO group and 57 U/L in the 8 × 10^{14} vg/kg KO group. This was statistically significant for the 8 × 10^{13} vg/kg KO group and not the 8 × 10^{14} vg/kg KO group in comparison with uninjected KOs. The mean for WTs injected with vector at 8 × 10^{14} vg/kg was 30 U/L, which was not statistically different from the uninjected WT group which had a mean of 48 U/L. Sample sizes for ALT analysis: 10 uninjected KOs, 6 KOs injected with 8 × 10^{13} vg/kg, 5 KOs injected with 8 × 10^{14} vg/kg, 5 WTs injected with 8 × 10^{14} vg/kg, and 12 uninjected WTs. Measurement of ALP showed a change from a mean of 701 U/L in uninjected KOs to 459 U/L in the 8 × 10^{13} vg/kg group and 378 U/L in the 8 × 10^{14} vg/kg group. In neither case was this difference statistically significant compared with uninjected KOs. Mean ALP for the 8 × 10^{14} vg/kg WT group was 192 U/L, which was not statistically different from the uninjected WT group which had a mean ALP of 226 U/L. Sample sizes for ALP analysis: 10 uninjected KOs, 5 KOs injected with 8 × 10^{13} vg/kg, 5 KOs injected with 8 × 10^{14} vg/kg, 5 WTs injected with 8 × 10^{14} vg/kg 10 uninjected WTs. Measurement of AST showed a change from a mean of 399 U/L in uninjected KOs to 133 U/L in the 8 × 10^{13} vg/kg group and 252 U/L in the 8 × 10^{14} vg/kg group. In neither case was this statistically significant compared with uninjected KOs. Mean ALP for the 8 × 10^{14} vg/kg WT group was 107 U/L, which was not statistically different from the uninjected WT group, which had a mean ALP of 95 U/L. Sample sizes for AST analysis: 10 uninjected KOs, 5 KOs injected with 8 × 10^{13} vg/kg, 4 KOs injected with 8 × 10^{14} vg/kg, 4 WTs injected with 8 × 10^{14} vg/kg, and 10 uninjected WTs. Statistics: Kruskal-Wallis test with multiple comparisons.

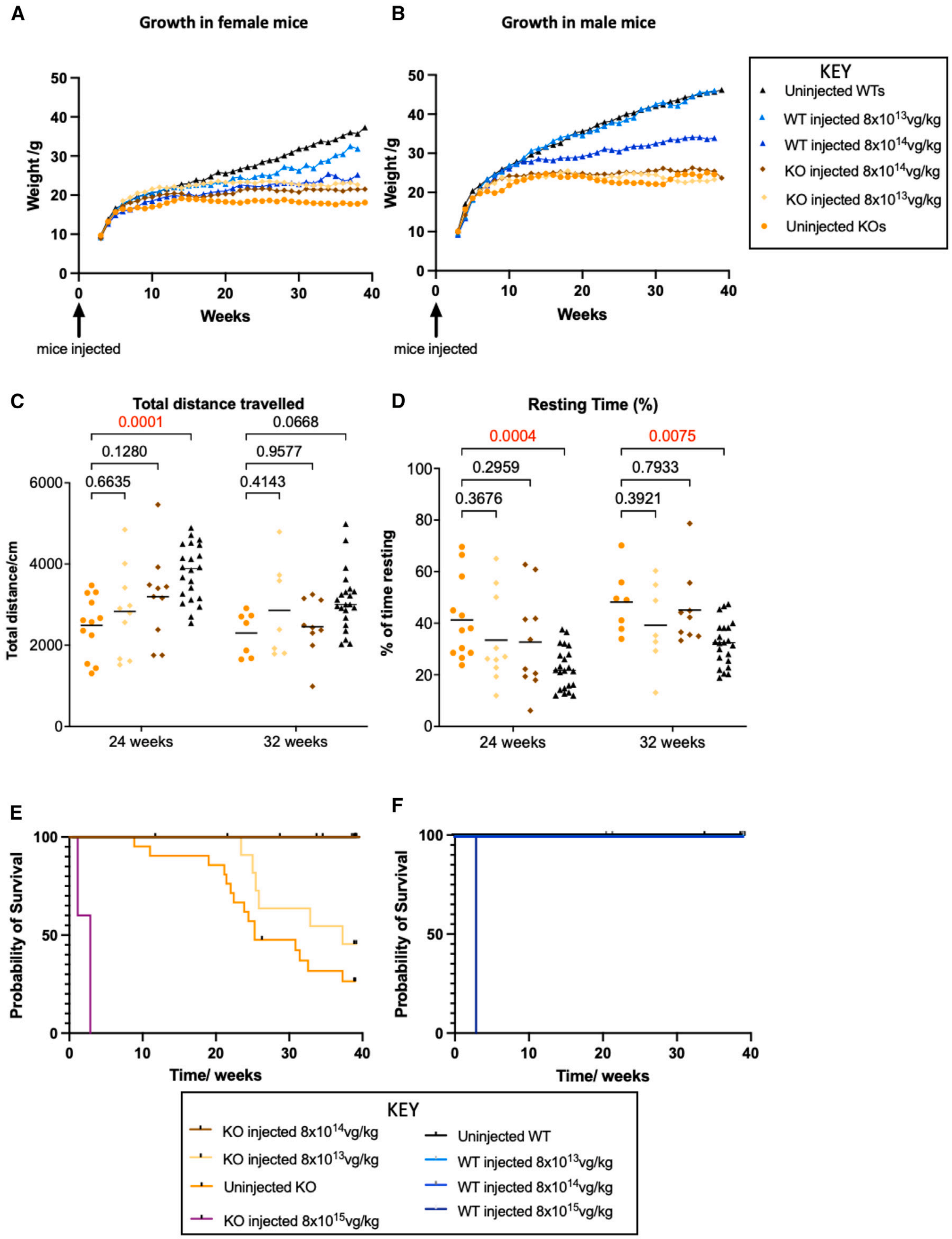

Figure 3. Growth, behavioral and survival outcomes following neonatal gene transfer

(A and B) Growth outcomes in female and male mice following neonatal gene transfer. Growth in KO mice was not restored to WT levels in either sex after gene therapy. Consistent improvement in weight compared with uninjected KO was seen for the female 8×10^{13} vg/kg group from 7 weeks onward (p = 0.01) and for the 8×10^{14} vg/kg group from 23 weeks onward (p = 0.0031). In male mice, neither the 8×10^{13} vg/kg nor the 8×10^{14} vg/kg group showed a consistent statistically significant difference in

(legend continued on next page)

significantly impaired in WT injected mice at the 8×10^{14} vg/kg dose, implying there was a negative impact on growth. No significant growth abnormality was seen in WTs injected at the 8×10^{13} vg/kg (Figures 3A and 3B). Locomotor abnormalities were not significantly improved in AAV9 injected KOs with respect to both total distance traveled and percentage resting time at both doses compared with uninjected WTs (Figures 3C and 3D). Survival data demonstrated a median survival of 177 days in uninjected KOs and 261 days in the 8×10^{13} vg/kg KO group, but there was no significant difference between the two (Mantel-Cox test, $p = 0.15$). However, for the 8×10^{14} vg/kg KO group, survival was significantly improved ($p = 0.0005$) with complete rescue in all injected animals to the end of the study at 42 weeks. WT mice injected at both 8×10^{13} vg/kg and 8×10^{14} vg/kg had 100% probability of survival in long-term follow-up. High-dose AAV9 (8×10^{15} vg/kg) caused early mortality in both WTs and KO mice with a median survival of 20 days, suggesting toxicity (Figures 3E and 3F).

DISCUSSION

Infantile-onset DGUOK deficiency is characterized by progressive liver failure, including conjugated hyperbilirubinemia, coagulopathy, transaminitis, tyrosinemia, and histological evidence of cholestasis, microsteatosis, fibrosis, hemochromatosis, cirrhosis, necrosis, portal hypertension, and hepatocellular carcinoma.[7–14] Management of DGUOK deficiency is supportive, including management of hypoglycemia, cholestasis, and the complications of liver failure.[15] Liver transplants in 14 patients showed unsatisfactory results: 1-year and 5-year post-transplant survival rates were 64% and 35%, respectively, which are lower than the average for liver transplantation for all causes.[16,17] These data and the shortage of suitable organ donors underscore a need for novel effective therapies. AAV-based gene therapy has been used to treat several mouse models of mitochondrial disorders.[18–24] This has included other genetic causes of MDDS for which liver targeting is needed, such as thymidine phosphorylase (TYMP) deficiency, for which rescue of plasma thymidine and deoxyuridine levels were achieved and MPV17 deficiency, for which liver mtDNA depletion was rescued.[23,24] Success in treating DGUOK deficiency with gene therapy may widen the scope for treating other genetic causes of MDDS. So far, the only clinical trials investigating the safety and efficacy of gene therapy for mitochondrial disorders are those targeting Leber hereditary optic neuropathy.[19]

Liver dysfunction is a core feature of DGUOK deficiency. Previous studies have shown that liver transgene expression after neonatal gene transfer in mice is short lived due to a dilutional effect as the liver grows.[25,26] In this work, long-term biodistribution studies demonstrated that KO mice had significantly higher GFP expression than WTs in liver. Islands of GFP-positive cells were seen in KO livers and the distribution of overall positivity seemed to be higher than that observed in the short term, contrary to expectations. These intriguing results raised the possibility that the AAV vector might be integrating into the host cell genome. AAV integration is a well-known phenomenon. Recent studies have suggested that the frequency of recombinant AAV integration may be higher than previously recognized, at a frequency of 1%–3% in liver.[27] The possibility that underlying mitochondrial dysfunction in KO mice may influence transgene expression also needs to be considered. One explanation could be that mitochondrial dysfunction leads to greater cell turnover in KO mice, which causes cell death. Transduced KO hepatocytes would be cured of their mitochondrial dysfunction and, therefore, could be conferred a survival advantage while non-transduced cells succumb to disease. Over time, cured cells could repopulate the liver resulting in higher VCN and GFP expression than that seen in WT mice injected at the same dose. Similar observations have been seen in another metabolic liver disease, fumarylacetoacetate hydrolase deficiency.[28] Concerns have been raised over potential oncogenic effects of AAV integration[29] and indeed one animal in this study injected with the 8×10^{14} vg/kg gene therapy dose developed hepatocellular carcinoma (HCC) in long-term follow-up. While it is possible that HCC could be gene therapy related, as has been demonstrated in other mouse preclinical gene therapy studies, it should be noted that HCC is also a recognized complication of liver disease in patients with DGUOK deficiency.[30] It is also noted that no causative correlation has been found in patients who have received AAV gene

weight compared with uninjected KOs. WT mice administered the gene therapy vector for toxicity studies demonstrated a statistically significant reduction in growth in female mice at 8×10^{14} vg/kg compared with uninjected WTs ($p = 0.0391$). Statistical comparison in male mice was not possible due to small sample size. Sample sizes for females: 12 uninjected KOs, 4 KOs injected with 8×10^{13} vg/kg KOs, 6 KOs injected with 8×10^{14} vg/kg KOs, 4 WTs injected with 8×10^{13} vg/kg, 5 WTs injected with 8×10^{14} vg/kg, and 9 uninjected WTs. Sample sizes for males: 9 uninjected KOs, 8 KOs injected with 8×10^{13} vg/kg, 5 KOs injected with 8×10^{14} vg/kg KO, 2 WTs injected with 8×10^{13} vg/kg, 1 WT injected with 8×10^{14} vg/kg, and 14 uninjected WTs. Statistics: mixed effects analysis with Dunnett's test for multiple comparisons. (C and D) Total distance traveled and percentage resting time in injected mice. Uninjected KOs demonstrated decreased total distance traveled compared with uninjected WTs. This was statistically significant at 24 weeks ($p = 0.0001$) but not at 32 weeks ($p = 0.0669$). There was a trend of improvement in total distance traveled in both of the injected KO groups but with much variability and no significant differences when compared with uninjected KOs. Uninjected KOs showed a significant increase in percentage resting time compared with uninjected WT mice at both 24 and 32 weeks ($p = 0.0004$ and 0.0075, respectively). The treated groups showed a trend of improved percentage resting time at both time points, but this was once again not statistically significant compared with uninjected KOs. Sample sizes at 24 weeks: 12 uninjected KOs, 10 KOs injected with 8×10^{13} vg/kg, 10 KOs injected with 8×10^{14} vg/kg, and 12 uninjected WTs. Sample sizes at 32 weeks: 7 uninjected KOs, 7 KOs injected with 8×10^{13} vg/kg, 9 KOs injected with 8×10^{14} vg/kg, and 22 uninjected WTs. Statistics: Kruskal-Wallis test with multiple comparisons. (E and F) Survival in injected KO and WT mice expressed as probability vs. time. Graphs show means, p values are indicated above the brackets. KOs injected with 8×10^{14} vg/kg showed no mortality in follow-up, which implied a significant improvement in survival at this dose ($p = 0.0005$). The 8×10^{13} vg/kg KO group had a median survival of 261 days; however, this was not statistically significant ($p = 0.15$). WT mice injected at 8×10^{14} vg/kg and 8×10^{13} vg/kg had no mortality. Both WT and KO mice injected at 8×10^{15} vg/kg had significantly reduced survival compared with uninjected WTs ($p < 0.0001$), with a median survival of 20 days. Sample sizes: 21 uninjected KOs, 12 KOs injected with 8×10^{13} vg/kg, 11 KOs injected with 8×10^{14} vg/kg, 5 KOs injected with 8×10^{15} vg/kg, 6 WTs injected with 8×10^{13} vg/kg, 6 WTs injected with 8×10^{14} vg/kg, and 7 WTs injected with 8×10^{15} vg/kg, 23 uninjected WTs. Statistics: Mantel-Cox test.

therapy in clinical trials to date.[31] Another possibility is that higher GFP expression in KO mice may not lie at the level of cellular transduction, but more distally at the level of RNA or protein expression. Indeed, we observed significantly higher transgene RNA expression in injected KOs compared with WTs delivered the same AAV dose.

Furthermore, the observation of higher transgene expression in injected KOs has dose implications, since the dose needed to achieve high long-term transgene expression could be lower than anticipated, which represents a major positive consideration given that toxicity is well recognized at high AAV doses. Other unanswered questions include whether other liver mitochondrial diseases may exhibit the same phenomenon seen in the DGUOK mouse model, and whether the same would be seen in humans. If this were true, then AAV9-based gene therapy could be a preferred treatment for mitochondrial diseases involving the liver.

The clinical definition of MDDS is less than 30% of healthy controls. The mouse model of DGUOK deficiency faithfully demonstrates liver mtDNA content less than 5% of WT levels, the same as those seen in infantile onset hepatocerebral MDDS in humans. Efficacy of liver-directed neonatal gene therapy was evaluated at AAV9 doses of 8×10^{13} vg/kg and 8×10^{14} vg/kg. VCN studies showed excellent dose-dependent transduction of liver in injected KO mice (1.57 and 5.03/cell for the 8×10^{13} vg/kg and 8×10^{14} vg/kg dose groups, respectively) corresponding with 79.2% and 99.3% cellular transduction. Mean *hDGUOK* RNA expression in injected KOs was approximately 100-fold higher than mean endogenous WT *mDguok* levels, in both the 8×10^{13} vg/kg and 8×10^{14} vg/kg groups suggesting saturation of RNA expression at this dose range. Therapeutic efficacy was observed in liver at 8×10^{14} vg/kg, restoring liver mtDNA content to 82% of WT levels, respectively. For the 8×10^{14} vg/kg group, there was no statistical difference compared with WTs, implying complete rescue. For the 8×10^{13} vg/kg group, the mean mtDNA copy number was 55% of WT levels but, despite this strong trend of improvement, there was no statistically significant difference to uninjected KOs. Statistically, these data seem to be skewed by a single outlier injected KO animal for which transduction in liver was low (VCN 0.26/cell) and mtDNA copy number remained low at 6% of WT levels.

Human and mouse *Dguok* cDNA have 80.6% identity, corresponding with 75.1% identity at an amino acid level; however, their 39-amino-acid-long mitochondrial targeting peptides, which enable uptake of DGUOK protein into mitochondria are quite dissimilar (approximately 48% identity). Therefore, it is possible that, even if hDGUOK protein were expressed proportionately to *hDGUOK* RNA, species differences in mitochondrial targeting could potentially lead to a decrease in hDGUOK localization to mitochondria. However, it was not possible to include DGUOK protein expression as an outcome measure due to the absence of a specific working antibody for DGUOK. Despite these species differences in amino acid sequences, we observed a significant amelioration of mtDNA depletion to well above the clinical definition (30% of WT controls). Rescue of mtDNA copy number in the injected KO groups helps to answer the question of what proportion of transduced hepatocytes is required to ensure sufficient rescue of mtDNA copy number to greater than 30% of WT levels. Our results suggest that approximately 35% transduced cells are needed to achieve a mtDNA content of 30% (data not shown).

Having demonstrated amelioration of mtDNA copy number in liver, we then proceeded to investigate whether OXPHOS abnormalities and transaminitis were rescued. We observed an improvement in complex I, III, and IV activities at both doses. The extent of rescue for the lower dose (complex I to 93% of WT levels, complex III to 81% of WT levels, complex IV to 66% of WT levels) is particularly noteworthy because it implied that a mean mtDNA copy number of 55% was sufficient to achieve significant rescue of liver mitochondrial dysfunction and subsequent amelioration of liver transaminases.

Patients with infantile-onset DGUOK deficiency also develop neurological diseases, including hypotonia, developmental delay, ptosis, rotatory nystagmus, and seizures. In the transplanted patient cohort, four patients had severe neurological progression despite liver transplantation, including one who had no apparent neurological disease before liver transplantation, implying that liver transplantation does not prevent or rescue neurological disease.[17] The DGUOK KO mouse model recapitulates the brain phenotype by exhibiting decreased mtDNA levels and complex IV deficiency. Although targeting of the liver through IV injections was the primary aim of this study, we also assessed brain targeting via this route. Long-term biodistribution studies demonstrated poor brain transduction in both WTs and KOs. In the gene therapy experiments, VCNs at either dose (8×10^{13} vg/kg or 8×10^{14} vg/kg) were low (0.05 and 0.09/cell, respectively) implying low transduction. It was, therefore, unsurprising that there was no significant improvement in mean mtDNA copy number or complex IV activity.

Alternative strategies or intracranial routes of delivery are needed to improve gene transfer to the brain. In another mouse model of MDDS caused by deficiency of the twinkle helicase (encoded by *Twnk*) that is associated with encephalopathic MDDS in humans, mtDNA depletion in glia specifically led to astrogliosis, analogous to our observations in the DGUOK-deficient mouse model.[32] These data suggest that adequate glial transduction is essential to rescue neurological involvement in this model.

Although we saw complete correction of liver disease, we did not observe rescue of growth. These data suggest that the involvement of other organs could contribute to poor weight gain, for example, the brain, kidney, skeletal muscle, and gastrointestinal or endocrine systems. Further work is needed to interrogate this. We also assessed locomotion in injected mice. The main abnormalities found in the KO strain were increased percent resting time and reduced total distance traveled. Neonatal gene transfer did not significantly improve total distance traveled and percent resting time. These data suggest that locomotor abnormalities in KOs may also have a multisystemic etiology.

Survival analyses showed complete rescue of survival at 8×10^{14} vg/kg and no significant improvement at the 8×10^{13} vg/kg dose. As liver transaminitis was significantly ameliorated at both doses, involvement of other organs in the model could explain these findings. One possibility is that skeletal muscle disease involvement negatively influences survival in this KO model. We observed that skeletal muscle mtDNA copy number and survival were both improved at the higher 8×10^{14} vg/kg dose but not at the 8×10^{13} vg/kg dose in injected KO mice. However, myopathic disease is not a prominent clinical feature of infantile-onset DGUOK deficiency. The data imply that reduced survival in this model may not be a clinically relevant outcome measure, if it is indeed, as we suspect, affected by the presence of skeletal muscle disease involvement.

We sought to clearly define an upper limit for dosing our AAV vector by using a dose of 8×10^{15} vg/kg in the highest dose group. This caused toxicity in both KO and WT mice resulting in early death. At the 8×10^{14} vg/kg or 8×10^{13} vg/kg doses, there was no increase in mortality seen in injected WT mice, but there was a decrease in growth at 8×10^{14} vg/kg. However, for the lower 8×10^{13} vg/kg dose group, growth was normal. These data suggest a dose-dependent toxic effect on growth in injected WTs. The mechanisms underlying this toxicity are unclear, since mtDNA copy number in liver, brain, or skeletal muscle, and blood liver function tests were normal in injected WTs. Nevertheless, it is clear that IV doses of 8×10^{14} vg/kg or greater are not safe. It is also important to note that from a clinical perspective, doses of greater than 1×10^{14} vg/kg have been associated with toxicity in gene therapy for other diseases.[33]

We aimed to also define the minimum efficacious dose needed to rescue liver disease. So far both the 8×10^{13} vg/kg and the 8×10^{14} vg/kg doses were able to improve mean liver mtDNA content to more than 30% of WT controls, and completely normalized ALT levels. Further IV dose de-escalations would be needed to ascertain the minimum efficacious dose for liver-directed gene therapy in this model. In the future, to build on this work, codon optimization of the transgene sequence to optimize RNA stability and translation may enable successful IV dose de-escalation without compromising efficacy. Considering the dosing requirements of the two main organs involved in DGUOK deficiency (the liver and brain), it is clear that achieving efficacious targeting of both organs using a single neonatal IV dose of this AAV9 construct will be challenging, since 8×10^{14} vg/kg was unable to transduce the brain sufficiently and higher doses seem to be toxic. Alternative AAV capsid configurations or intracranial routes of delivery need to be considered to achieve efficacy and safety.

MATERIALS AND METHODS

Cloning of plasmids

Two payload plasmids were generated, pAAV-CAG-intron-*hDGUOK*-T2A-eGFP-WPRE-bGH-pA and pAAV-CAG-intron-*hDGUOK*-WPRE-bGH-pA, for biodistributional and gene therapy studies, respectively. First, pAAV-CMV-GFP-WPRE-bGH-pA (University of Pennsylvania) was linearized by PCR (primer sequences available on request). Gene blocks containing *hDGUOK-T2A and hDGUOK* were obtained from Integrated DNA technologies (IDT, Leuven, Belgium). The CAG promoter and intron were restriction digested from existing plasmids. Ligation of the various components was undertaken using an In-Fusion cloning kit (Takara Bio-Europe, Paris, France). The ligation reaction was transformed into Stellar-competent cells as per manufacturer protocols. Cells were incubated on LB/agar plates overnight and clones selected. The DNA sequences obtained from clones were confirmed using Sanger sequencing.

AAV9 vector production and titration

DNA was amplified for AAV production using an Invitrogen maxiprep kit. Helper and AAV9 plasmids (Harvard University and University of Pennsylvania, respectively) were used in AAV production using a triple transfection approach in HEK293T cells, followed by high-performance liquid chromatography (HPLC) purification (AKTA prime) and vector concentration as described previously.[34] Following DNAse treatment, the vector was titrated via qPCR using Luna SYBR green reagents (New England Biolabs, Ipswich, MA, USA) as per manufacturer recommendations. Primer sequences targeting *hDGUOK* were used for titration and are available on request. qPCR standards were made up using gene blocks as above.

Animal experiments

Husbandry

Experimental animals were maintained at an experimental animal facility in adherence with Animal Research: Reporting of In Vivo Experiments (ARRIVE) guidelines and UK Home Office regulations. Experiments were approved by UCL Biological Services. Mice were housed in individually ventilated cages, subject to day/night light cycles, provided with drinking water, standard laboratory rodent chow, and nesting materials.

Breeding

Adult *Dguok*$^{+/-}$ mice were maintained on an albino C57BL/6N background for breeding of *Dguok*$^{-/-}$ mice (KOs) which were identified by genotyping as described previously.[6]

Behavioral testing

Open field testing was carried out using Harvard Panlab equipment (Barcelona, Spain) in 25 cm × 25 cm square arenas. Recordings of 10 min duration were taken in moderate lighting. Data were analyzed using SMART v3 software. For grip strength testing animals were placed onto a 1 cm × 1 cm metal grid and then gently inverted over a large transparent plastic box. The time taken to fall in seconds was recorded (average of three attempts taken as the final measurement).

IV injections

Injections were performed within the first 48 h of life via the superficial temporal vein using a 33G Hamilton needle. We administered 20 μL of vector per pup. Investigators undertaking gene therapy experiments were blinded as to which animals received injections and animals were randomized to treatment groups. Both male and female mice were used. In initial biodistribution studies, WTs were injected

at birth with AAV9-CAG-*hDGUOK*-GFP at 3×10^{13} vg/kg or 3×10^{14} vg/kg and followed up for 6 weeks. Tissues were collected and stained for GFP. We assessed long-term GFP expression in both WTs and KOs injected with AAV9-CAG-*hDGUOK*-GFP at 3×10^{14} vg/kg only and followed up to 9 months or the humane endpoint, whichever was sooner, to determine longevity of transgene expression by stereoscopic microscopy, anti-GFP immuno-histochemistry, and VCN studies.

Gene therapy studies were undertaken in neonatal KO mice, using the gene therapy vector, AAV9-CAG-*hDGUOK*. WT littermates were also injected for toxicity studies. The doses used were 8×10^{13} vg/kg, 8×10^{14} vg/kg, and 8×10^{15} vg/kg. Animals were followed until 9 months or the humane endpoint, whichever was earlier.

Collection and processing of tissues

For blood sampling and immunohistochemistry studies, animals were anesthetized using isoflurane. Blood was collected via the intracardiac route and whole-body perfusion undertaken with 1× PBS. Serum samples were obtained after centrifugation of clotted blood at 13,500 rpm for 6 min. Tissues were fixed in 4% paraformaldehyde for 48 h, transferred to 30% sucrose, then sectioned to 40-nm sections using a microtome (Epredia, Kalamazoo, MI, USA) and stored at 4°C in TBSAF. Tissues for OXPHOS studies were obtained by cervical dislocation without anesthesia and snap frozen in dry ice. Homogenates were prepared for OXPHOS studies as described previously.[32] DNA and RNA were extracted using Qiagen DNeasy and Invitrogen RNA extraction kits as per manufacturer protocols.

Droplet digital PCR

Tissue VCN and mtDNA copy numbers were determined using droplet digital PCR (ddPCR). The targets used were *hDGUOK* and *Mt-Nd1*, with *Rpp30* as the reference (Bio-Rad [Hercules, CA, USA]assay catalog numbers 10042958 and 10042961, respectively). Primers and probe sequences for *hDGUOK* are available on request. Samples were first prepared by restriction enzyme digestion with *HaeIII* (NEB) for 1 h at 37°C followed by ddPCR using a Bio-Rad Auto DG droplet generator. Thermocycler settings were: initial activation 95°C 10 min, denaturation 94°C 30 s, annealing extension 55.8°C 1 min, cycles 40, deactivation 98°C 10 min, 4°C hold. Samples were then read by the Bio-Rad droplet reader and analyzed using QuantaSoft software v1.7 regulatory edition.

qPCR

qPCR was performed to evaluate RNA expression, utilizing *hDGUOK* and *mDguok* targets, with *mGapdh* as the reference and NEB Luna mastermix for probes. Final concentrations were 450 nm for target primers and probes and 250 nM for *mGapdh* primers and probe. Primer and probe sequences are available on request. qPCR standards were made up using Gene blocks obtained from IDT for all three targets and were run alongside each qPCR plate. Samples were run in triplicate. Thermocycler settings: initial activation 50°C 2 min, initial denaturation 95°C 10 min, denaturation 95°C 15 s, annealing/extension 60°C 1 min, cycles 40, hold 4°C. Final expression data were expressed as a ratio to *mGapdh* expression.

OXPHOS studies

OXPHOS studies (complexes I, II + III, III, IV, and citrate synthase as a reference enzyme) were undertaken as previously described.[35–39]

Immunohistochemistry and microscopy

Immunohistochemical staining for GFAP-positive astrocytes and CD68-positive microglia was undertaken to investigate the possibility of astrogliosis and microgliosis in baseline phenotyping. Anti-GFP immunohistochemistry was used to assess biodistribution. Free-floating staining of brain and visceral organs was undertaken as previously described.[40] Primary antibodies used: rabbit anti-GFP for GFP staining (Abcam [Cambridge, UK], dilution 1:10,000), mouse anti-GFAP for GFAP staining (0 & Co. Rhaway, NJ, USA, 1:1,000), and rat anti-CD68 for CD68 staining (Bio-Rad, 1:100). Secondary antibodies used: goat anti-rabbit for GFP staining (1:1,000), goat anti-mouse for GFAP staining (1:1,000) and rabbit anti-rat for CD68 staining (1:1,000) (all from Vector Laboratories, Burlingame, CA, USA). A 3,3′-diaminobenzidine reporter was used.

Blood tests

An NX600 Dri-chem analyzer (Fujifilm) was used to measure blood liver function tests (ALT, AST, ALP, albumin, and bilirubin) and blood glucose from serum as per manufacturer recommendations. Ammonia was measured from whole blood using a NX10N analyzer (Fujifilm) as per manufacturer recommendations. Amino acids were analyzed as phenylisothiocyanate derivatives by reverse-phase HPLC using an ODS-bonded silica column (Waters WAT010950) and UV detection at 254 nm, based on previously reported methods.[41,42]

Statistical analysis

GraphPad prism software v9 was used for statistical analysis. A p value of less than 0.05 was considered statistically significant. Parametric or non-parametric tests were used to compare groups depending on whether data were normally distributed. Statistical tests used in each analysis are indicated in the figure captions. Multiple comparisons correction was used in all cases where this was relevant. For survival analyses, the Mantel-Cox test was used.

DATA AND CODE AVAILABILITY

Datasets will be made available on request.

ACKNOWLEDGMENTS

N.K. received an Action Medical Research Clinical Research Training Fellowship award GN2682 to undertake this work. S.R. acknowledges grant funding from Great Ormond Street Hospital Children's Charity, the Lily Foundation, and the National Institute for Health Research (NIHR) Great Ormond Street Hospital Biomedical Research Centre. S.W received support from MRC grant MR/T016809/1, Action Medical Research grant GN2647, Action Medical Research grant GN2984, and the Wellbeing of Women. R.K. received support from LifeArc grant P2020-0008 and Great Ormond Street Hospital Children's Charity grant V4720. J.A.D. received support from LifeArc grant P2020-0008. R.P. was funded through a UCL School of Life and Medical Science Impact PhD Studentship. A.K. was funded by Karolinska Institute grant 15–0953; Swedish Cancer Society grant CAN 2016/1342-1345; and Swedish Research Council grant K2014-66X12162-18-3.

The views expressed are those of the authors and not necessarily those of the NHS, the NIHR, or the Department of Health.

AUTHOR CONTRIBUTIONS

Conceptualization: N.K., R.K., S.W., S.R., and J.C. Experiments: N.K., M.G., H.P., R.K., S.W., J.D., R.P., and N.S. Drafting manuscript and figures: N.K. Review and editing of manuscript: all authors.

DECLARATION OF INTERESTS

S.W. is a founder of and consultant for Bloomsbury Genetic Therapies and is a member of the SMAB of Forge Biologics. S.R. is a member of the SAB for Khondrion, and has provided consultancy on primary mitochondrial diseases for pharmaceutical companies including BioMedical Insights, Neurovive, Partners4Access, Pfizer, Epirium, Stealth Biotherapeutics, Taysha Gene Therapies, Modis Therapeutics, Pretzel Therapeutics, Access Infinity, Reneo, Glycomine, and Market Modelers. S.R. is Editor-in-Chief of the *Journal of Inherited Metabolic Disease* and *JIMD Reports*, Medical advisor to the Lily Foundation and the Freya Foundation, and special advisor to the UK Human Fertilisation and Embryology Authority. S.R. is an Executive Editor of the North American Metabolic Academy, a member of the Medical Research Council Clinical Training Panel, and sits on the Council of the Society for the Study of Inborn Errors of Metabolism.

SUPPLEMENTAL INFORMATION

Supplemental information can be found online at https://doi.org/10.1016/j.omtm.2024.101397.

REFERENCES

1. Viscomi, C., and Zeviani, M. (2017). MtDNA-maintenance defects: syndromes and genes. J. Inherit. Metab. Dis. *40*, 587–599. https://doi.org/10.1007/s10545-017-0027-5.
2. Moraes, C.T., Shanske, S., Tritschler, H.J., Aprille, J.R., Andreetta, F., Bonilla, E., Schon, E.A., and DiMauro, S. (1991). mtDNA depletion with variable tissue expression: a novel genetic abnormality in mitochondrial diseases. Am. J. Hum. Genet. *48*, 492–501.
3. Sezer, T., Ozçay, F., Balci, O., and Alehan, F. (2015). Novel deoxyguanosine kinase gene mutations in the hepatocerebral form of mitochondrial DNA depletion syndrome. J. Child. Neurol. *30*, 124–128. https://doi.org/10.1177/0883073813517000.
4. Johansson, M., and Karlsson, A. (1996). Cloning and expression of human deoxyguanosine kinase cDNA. Proc. Natl. Acad. Sci. USA *93*, 7258–7262. https://doi.org/10.1073/pnas.93.14.7258.
5. Saada, A. (2008). Mitochondrial deoxyribonucleotide pools in deoxyguanosine kinase deficiency. Mol. Genet. Metabol. *95*, 169–173. https://doi.org/10.1016/j.ymgme.2008.07.007.
6. Zhou, X., Curbo, S., Zhao, Q., Krishnan, S., Kuiper, R., and Karlsson, A. (2019). Severe mtDNA depletion and dependency on catabolic lipid metabolism in DGUOK knockout mice. Hum. Mol. Genet. *28*, 2874–2884. https://doi.org/10.1093/hmg/ddz103.
7. Labarthe, F., Dobbelaere, D., Devisme, L., De Muret, A., Jardel, C., Taanman, J.W., Gottrand, F., and Lombès, A. (2005). Clinical, biochemical and morphological features of hepatocerebral syndrome with mitochondrial DNA depletion due to deoxyguanosine kinase deficiency. J. Hepatol. *43*, 333–341. https://doi.org/10.1016/j.jhep.2005.03.023.
8. Mandel, H., Szargel, R., Labay, V., Elpeleg, O., Saada, A., Shalata, A., Anbinder, Y., Berkowitz, D., Hartman, C., Barak, M., et al. (2001). The deoxyguanosine kinase gene is mutated in individuals with depleted hepatocerebral mitochondrial DNA. Nat. Genet. *29*, 337–341. https://doi.org/10.1038/ng746.
9. Dimmock, D.P., Zhang, Q., Dionisi-Vici, C., Carrozzo, R., Shieh, J., Tang, L.Y., Truong, C., Schmitt, E., Sifry-Platt, M., Lucioli, S., et al. (2008). Clinical and molecular features of mitochondrial DNA depletion due to mutations in deoxyguanosine kinase. Hum. Mutat. *29*, 330–331. https://doi.org/10.1002/humu.9519.
10. Hanchard, N.A., Shchelochkov, O.A., Roy, A., Wiszniewska, J., Wang, J., Popek, E.J., Karpen, S., Wong, L.J.C., and Scaglia, F. (2011). Deoxyguanosine kinase deficiency presenting as neonatal hemochromatosis. Mol. Genet. Metabol. *103*, 262–267. https://doi.org/10.1016/j.ymgme.2011.03.006.
11. Pronicka, E., Węglewska-Jurkiewicz, A., Taybert, J., Pronicki, M., Szymańska-Dębińska, T., Karkucińska-Więckowska, A., Jakóbkiewicz-Banecka, J., Kowalski, P., Piękutowska-Abramczuk, D., Pajdowska, M., et al. (2011). Post mortem identification of deoxyguanosine kinase (DGUOK) gene mutations combined with impaired glucose homeostasis and iron overload features in four infants with severe progressive liver failure. J. Appl. Genet. *52*, 61–66. https://doi.org/10.1007/s13353-010-0008-y.
12. Kasapkara, C.S., Tümer, L., Küçükçongar, A., Hasanoğlu, A., Seneca, S., and De Meirleir, L. (2013). DGUOK-related mitochondrial DNA depletion syndrome in a child with an early diagnosis of glycogen storage disease. J. Pediatr. Gastroenterol. Nutr. *57*, e28–e29. https://doi.org/10.1097/MPG.0b013e31826bd4ed.
13. Al-Hussaini, A., Faqeih, E., El-Hattab, A.W., Alfadhel, M., Asery, A., Alsaleem, B., Bakhsh, E., Ali, A., Alasmari, A., Lone, K., et al. (2014). Clinical and molecular characteristics of mitochondrial DNA depletion syndrome associated with neonatal cholestasis and liver failure. J. Pediatr. *164*, 553–559.e92. https://doi.org/10.1016/j.jpeds.2013.10.082.
14. Ünal, O., Hismi, B., Kilic, M., Gulsen, H.H., Coskun, T., Sivri, S.H., Dursun, A., Yuce, A., and Tokatli, A. (2017). Deoxyguanosine kinase deficiency: a report of four patients. J. Pediatr. Endocrinol. Metab. *30*, 697–702. https://doi.org/10.1515/jpem-2016-0268.
15. El-Hattab, A.W., Scaglia, F., and Wong, L.J. (1993). Deoxyguanosine Kinase Deficiency. In GeneReviews((R)), M.P. Adam, H.H. Ardinger, R.A. Pagon, S.E. Wallace, L.J.H. Bean, K. Stephens, and A. Amemiya, eds.
16. Dimmock, D.P., Dunn, J.K., Feigenbaum, A., Rupar, A., Horvath, R., Freisinger, P., Mousson de Camaret, B., Wong, L.J., and Scaglia, F. (2008). Abnormal neurological features predict poor survival and should preclude liver transplantation in patients with deoxyguanosine kinase deficiency. Liver. Transpl. *14*, 1480–1485. https://doi.org/10.1002/lt.21556.
17. Grabhorn, E., Tsiakas, K., Herden, U., Fischer, L., Freisinger, P., Marquardt, T., Ganschow, R., Briem-Richter, A., and Santer, R. (2014). Long-term outcomes after liver transplantation for deoxyguanosine kinase deficiency: a single-center experience and a review of the literature. Liver Transplant. *20*, 464–472. https://doi.org/10.1002/lt.23830.
18. Di Meo, I., Auricchio, A., Lamperti, C., Burlina, A., Viscomi, C., and Zeviani, M. (2012). Effective AAV-mediated gene therapy in a mouse model of ethylmalonic encephalopathy. EMBO Mol. Med. *4*, 1008–1014. https://doi.org/10.1002/emmm.201201433.
19. Biousse, V., Newman, N.J., Yu-Wai-Man, P., Carelli, V., Moster, M.L., Vignal-Clermont, C., Klopstock, T., Sadun, A.A., Sergott, R.C., Hage, R., et al. (2021). Long-Term Follow-Up After Unilateral Intravitreal Gene Therapy for Leber Hereditary Optic Neuropathy: The RESTORE Study. J. Neuro Ophthalmol. *41*, 309–315. https://doi.org/10.1097/WNO.0000000000001367.
20. Bouaita, A., Augustin, S., Lechauve, C., Cwerman-Thibault, H., Bénit, P., Simonutti, M., Paques, M., Rustin, P., Sahel, J.A., and Corral-Debrinski, M. (2012). Downregulation of apoptosis-inducing factor in Harlequin mice induces progressive and severe optic atrophy which is durably prevented by AAV2-AIF1 gene therapy. Brain *135*, 35–52. https://doi.org/10.1093/brain/awr290.
21. Cabrera-Pérez, R., Vila-Julià, F., Hirano, M., Mingozzi, F., Torres-Torronteras, J., and Marti, R. (2019). Alpha-1-Antitrypsin Promoter Improves the Efficacy of an Adeno-Associated Virus Vector for the Treatment of Mitochondrial Neurogastrointestinal Encephalomyopathy. Hum. Gene. Ther. *30*, 985–998. https://doi.org/10.1089/hum.2018.217.
22. Di Meo, I., Marchet, S., Lamperti, C., Zeviani, M., and Viscomi, C. (2017). AAV9-based gene therapy partially ameliorates the clinical phenotype of a mouse model of Leigh syndrome. Gene. Ther. *24*, 661–667. https://doi.org/10.1038/gt.2017.53.
23. Lopez-Gomez, C., Sanchez-Quintero, M.J., Lee, E.J., Kleiner, G., Tadesse, S., Xie, J., Akman, H.O., Gao, G., and Hirano, M. (2021). Synergistic Deoxynucleoside and Gene Therapies for Thymidine Kinase 2 Deficiency. Ann. Neurol. *90*, 640–652. https://doi.org/10.1002/ana.26185.
24. Bottani, E., Giordano, C., Civiletto, G., Di Meo, I., Auricchio, A., Ciusani, E., Marchet, S., Lamperti, C., d'Amati, G., Viscomi, C., and Zeviani, M. (2014). AAV-mediated liver-specific MPV17 expression restores mtDNA levels and prevents diet-induced liver failure. Mol. Ther. *22*, 10–17. https://doi.org/10.1038/mt.2013.230.

25. Cunningham, S.C., Dane, A.P., Spinoulas, A., and Alexander, I.E. (2008). Gene Delivery to the Juvenile Mouse Liver Using AAV2/8 Vectors. Mol. Ther. *16*, 1081–1088. https://doi.org/10.1038/mt.2008.72.

26. Nathwani, A.C., Rosales, C., McIntosh, J., Rastegarlari, G., Nathwani, D., Raj, D., Nawathe, S., Waddington, S.N., Bronson, R., Jackson, S., et al. (2011). Long-term safety and efficacy following systemic administration of a self-complementary AAV vector encoding human FIX pseudotyped with serotype 5 and 8 capsid proteins. Mol. Ther. *19*, 876–885. https://doi.org/10.1038/mt.2010.274.

27. Dalwadi, D.A., Calabria, A., Tiyaboonchai, A., Posey, J., Naugler, W.E., Montini, E., and Grompe, M. (2021). AAV integration in human hepatocytes. Mol. Ther. *29*, 2898–2909. https://doi.org/10.1016/j.ymthe.2021.08.031.

28. Strom, S.C., Davila, J., and Grompe, M. (2010). Chimeric mice with humanized liver: tools for the study of drug metabolism, excretion, and toxicity. Methods. Mol. Biol. *640*, 491–509. https://doi.org/10.1007/978-1-60761-688-7_27.

29. Chandler, R.J., Sands, M.S., and Venditti, C.P. (2017). Recombinant Adeno-Associated Viral Integration and Genotoxicity: Insights from Animal Models. Hum. Gene. Ther. *28*, 314–322. https://doi.org/10.1089/hum.2017.009.

30. Keshavan, N., and Rahman, S. (2024). Natural history of deoxyguanosine kinase deficiency. Mol. Genet. Metab. *143*, 108554. https://doi.org/10.1016/j.ymgme.2024.108554.

31. Baruteau, J., Brunetti-Pierri, N., and Gissen, P. (2024). Liver-directed gene therapy for inherited metabolic diseases. J. Inherit. Metab. Dis. *47*, 9–21. https://doi.org/10.1002/jimd.12709.

32. Ignatenko, O., Chilov, D., Paetau, I., de Miguel, E., Jackson, C.B., Capin, G., Paetau, A., Terzioglu, M., Euro, L., and Suomalainen, A. (2018). Loss of mtDNA activates astrocytes and leads to spongiotic encephalopathy. Nat. Commun. *9*, 70. https://doi.org/10.1038/s41467-017-01859-9.

33. Chand, D., Mohr, F., McMillan, H., Tukov, F.F., Montgomery, K., Kleyn, A., Sun, R., Tauscher-Wisniewski, S., Kaufmann, P., and Kullak-Ublick, G. (2021). Hepatotoxicity following administration of onasemnogene abeparvovec (AVXS-101) for the treatment of spinal muscular atrophy. J. Hepatol. *74*, 560–566. https://doi.org/10.1016/j.jhep.2020.11.001.

34. Binny, C.J., and Nathwani, A.C. (2012). Vector systems for prenatal gene therapy: principles of adeno-associated virus vector design and production. Methods Mol. Biol. *891*, 109–131. https://doi.org/10.1007/978-1-61779-873-3_6.

35. Hargreaves, P., Rahman, S., Guthrie, P., Taanman, J.W., Leonard, J.V., Land, J.M., and Heales, S.J.R. (2002). Diagnostic value of succinate ubiquinone reductase activity in the identification of patients with mitochondrial DNA depletion. J. Inherit. Metab. Dis. *25*, 7–16. https://doi.org/10.1023/a:1015104910239.

36. Shepherd, D., and Garland, P.B. (1969). The kinetic properties of citrate synthase from rat liver mitochondria. Biochem. J. *114*, 597–610. https://doi.org/10.1042/bj1140597.

37. King, T.E. (1967). Preparations of succinate—cytochrome c reductase and the cytochrome b-c1 particle, and reconstitution of succinate-cytochrome c reductase. Meth. Enzymol. *10*, 216–225. https://doi.org/10.1016/0076-6879(67)10043-8.

38. Wharton, D.C., and Tzagoloff, A. (1967). Cytochrome oxidase from beef heart mitochondria. Methods Enzymol. *10*, 245–250. https://doi.org/10.1016/0076-6879(67)10048-7.

39. Ragan, C.I., T.W., M., Darley-Usmar, V.M., and Lowe, P.N. (1987). In Mitochondria: A Practical Approach, D.R.V.M. Darley-Usmar and M.T. Wilson, eds. (IRL Press)), pp. 79–113.

40. Baruteau, J., Perocheau, D.P., Hanley, J., Lorvellec, M., Rocha-Ferreira, E., Karda, R., Ng, J., Suff, N., Diaz, J.A., Rahim, A.A., et al. (2018). Argininosuccinic aciduria fosters neuronal nitrosative stress reversed by Asl gene transfer. Nat. Commun. *9*, 3505. https://doi.org/10.1038/s41467-018-05972-1.

41. Cohen, S.A., Bidlingmeyer, B.A., and Tarvin, T.L. (1986). PITC derivatives in amino acid analysis. Nature *320*, 769–770. https://doi.org/10.1038/320769a0.

42. Pramanik, B.C., Moomaw, C.R., Evans, C.T., Cohen, S.A., and Slaughter, C.A. (1989). Identification of phenylthiocarbamyl amino acids for compositional analysis by thermospray liquid chromatography/mass spectrometry. Anal. Biochem. *176*, 269–277. https://doi.org/10.1016/0003-2697(89)90308-4.

OMTM, Volume *33*

Supplemental information

Gene therapy prevents hepatic mitochondrial dysfunction in murine deoxyguanosine kinase deficiency

Nandaki Keshavan, Miriam Greenwood, Helen Prunty, Juan Antinao Diaz, Riccardo Privolizzi, John Counsell, Anna Karlsson, Neil Sebire, Simon Waddington, Rajvinder Karda, and Shamima Rahman

Figure S1

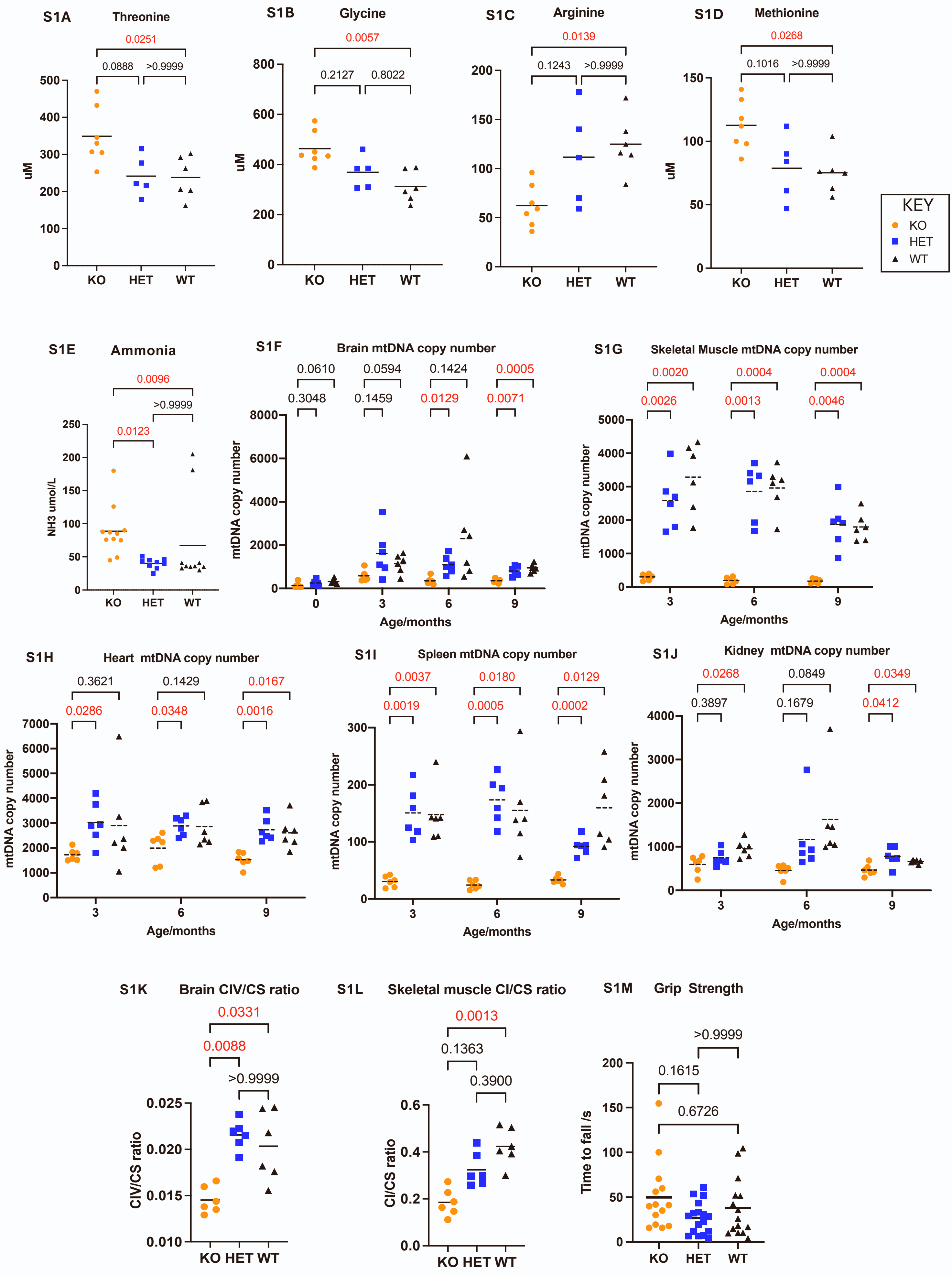
S1A Threonine
0.0251
0.0888 >0.9999
uM
KO HET WT
S1B Glycine
0.0057
0.2127 0.8022
S1C Arginine
0.0139
0.1243 >0.9999
S1D Methionine
0.0268
0.1016 >0.9999
KEY
KO
HET
WT
S1E Ammonia
0.0096
>0.9999
0.0123
NH3 umol/L
S1F Brain mtDNA copy number
0.0610 0.0594 0.1424 0.0005
0.3048 0.1459 0.0129 0.0071
mtDNA copy number
Age/months
S1G Skeletal Muscle mtDNA copy number
0.0020 0.0004 0.0004
0.0026 0.0013 0.0046
S1H Heart mtDNA copy number
0.3621 0.1429 0.0167
0.0286 0.0348 0.0016
S1I Spleen mtDNA copy number
0.0037 0.0180 0.0129
0.0019 0.0005 0.0002
S1J Kidney mtDNA copy number
0.0268 0.0849 0.0349
0.3897 0.1679 0.0412
S1K Brain CIV/CS ratio
0.0331
0.0088
>0.9999
CIV/CS ratio
S1L Skeletal muscle CI/CS ratio
0.0013
0.1363
0.3900
CI/CS ratio
S1M Grip Strength
>0.9999
0.1615
0.6726
Time to fall /s

Figure S1 Additional baseline phenotyping data for Dguok KO mouse model

S1A-S1D: Serum amino acid measurements for threonine, glycine, arginine and methionine. Threonine, glycine and methionine were significantly increased whereas arginine was reduced in KO mice compared to WTs. Sample sizes: 7KO, 5HET, 6WT. Statistics: Kruskal-Wallis test for multiple comparisons. **S1E:** Blood ammonia measurements at 9 months. Hyperammonaemia was demonstrated in KOs compared to HET and WT mice. Sample sizes: 11KO, 9HET, 10WT. Statistics: Kruskal-Wallis test for multiple comparisons. **S1F:** Brain mtDNA quantitation. KOs showed significant brain mtDNA depletion compared to WTs at 9 months but not prior to this time point. Sample size: 6 per genotype per time point. Statistics: 2-way ANOVA with Tukey's test for multiple comparisons. **S1G:** Skeletal muscle mtDNA quantitation. Skeletal muscle mtDNA depletion was demonstrated from 3 months onward. Sample size: 6 per genotype per time point. Statistics: 2-way ANOVA with Tukey's test for multiple comparisons. **S1H** Heart mtDNA quantitation. Heart mtDNA deficiency was demonstrated at 9 months. Sample size: 6 per genotype per time point. Statistics: 2-way ANOVA with Tukey's test for multiple comparisons. **S1I:** Spleen mtDNA quantitation. Spleen mtDNA depletion was demonstrated from 3 months onward. Sample size: 6 per genotype per time point. Statistics: 2-way ANOVA with Tukey's test for multiple comparisons. **S1J** Kidney mtDNA quantitation. Kidney mtDNA deficiency was demonstrated at 9 months. Sample size: 6 per genotype per time point. Statistics: 2-way ANOVA with Tukey's test for multiple comparisons. **S1K:** Brain complex IV/CS ratio at 9 months. KOs showed significant brain complex IV deficiency. Sample size: 6 per genotype. Statistics: Kruskal-Wallis test for multiple comparisons. **S1L:** Skeletal muscle complex I/CS ratio at 9 months. KOs showed skeletal muscle complex I deficiency. Sample size: 6 per genotype. Statistics: Kruskal-Wallis test for multiple comparisons. **S1M:** Grip strength testing undertaken as part of behavioural testing. No significant differences were seen between KOs and WT/HETs. Sample sizes: 14KO, 17HET, 15WT. Statistics: Kruskal-Wallis test for multiple comparisons. p values are indicated above the brackets. p values <0.05 are indicated in red.

Figure S2

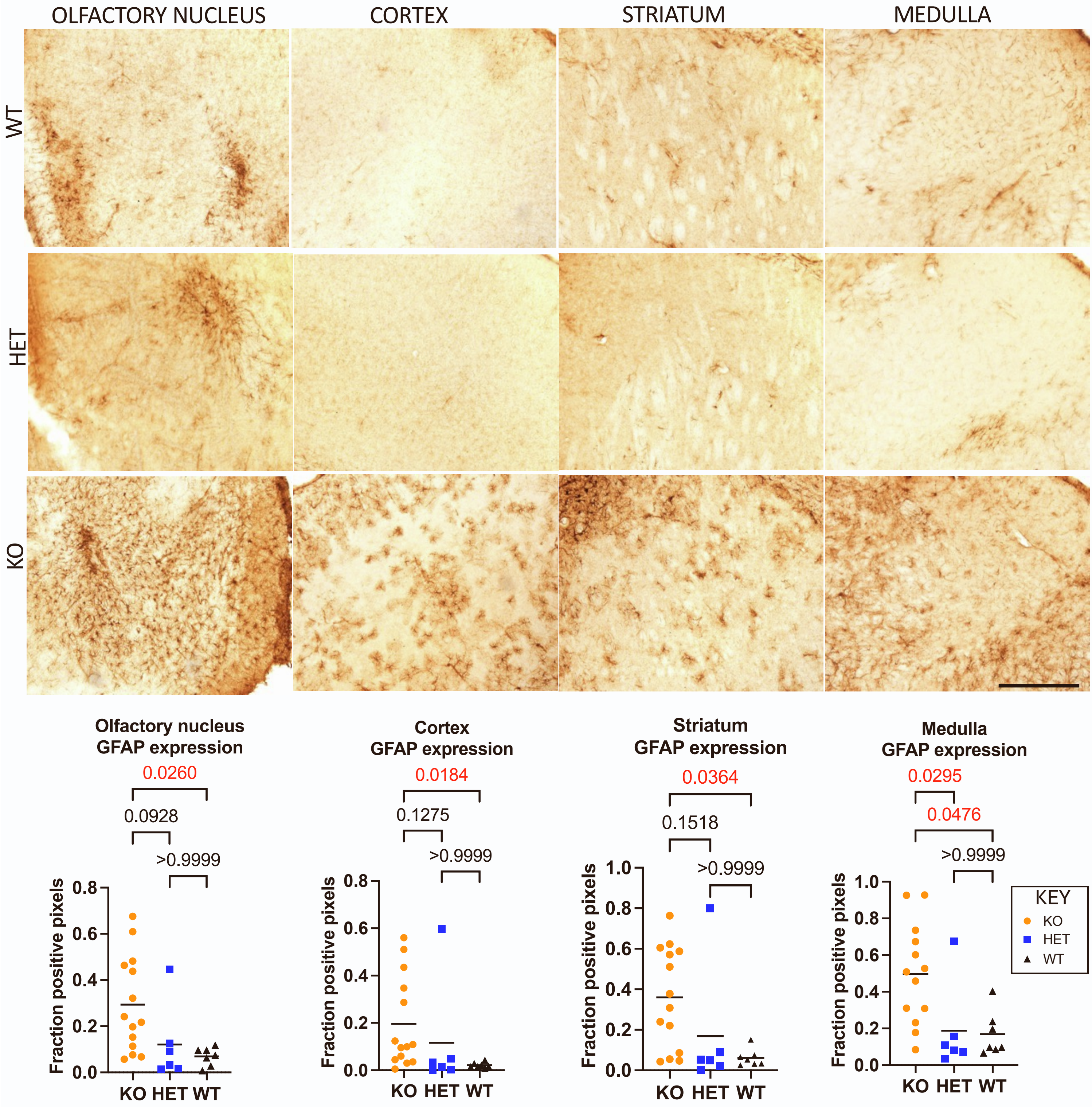

Figure S2 Characterisation of neurological phenotype in murine *Dguok* KO model. Neuroinflammatory phenotyping demonstrated widespread neuroinflammation in *Dguok* KO mice at 9 months. Astrogliosis was evident on anti-GFAP immunohistochemistry in diffuse brain regions (olfactory nucleus, cortex, striatum and medulla oblongata) of KO mice. Images displayed are representative of the mean expression for each genotype and were taken at x10 magnification, scale bar: 500µm. Graphs show quantitation of staining as determined by thresholding. Sample size: 7 WT, 6 HET, 14 KOs for olfactory nucleus, cortex, striatum and 7 WT, 6 HET, 13 KOs for medulla. Statistics: Kruskal Wallis test with multiple comparisons. p values <0.05 are highlighted red.

Figure S3

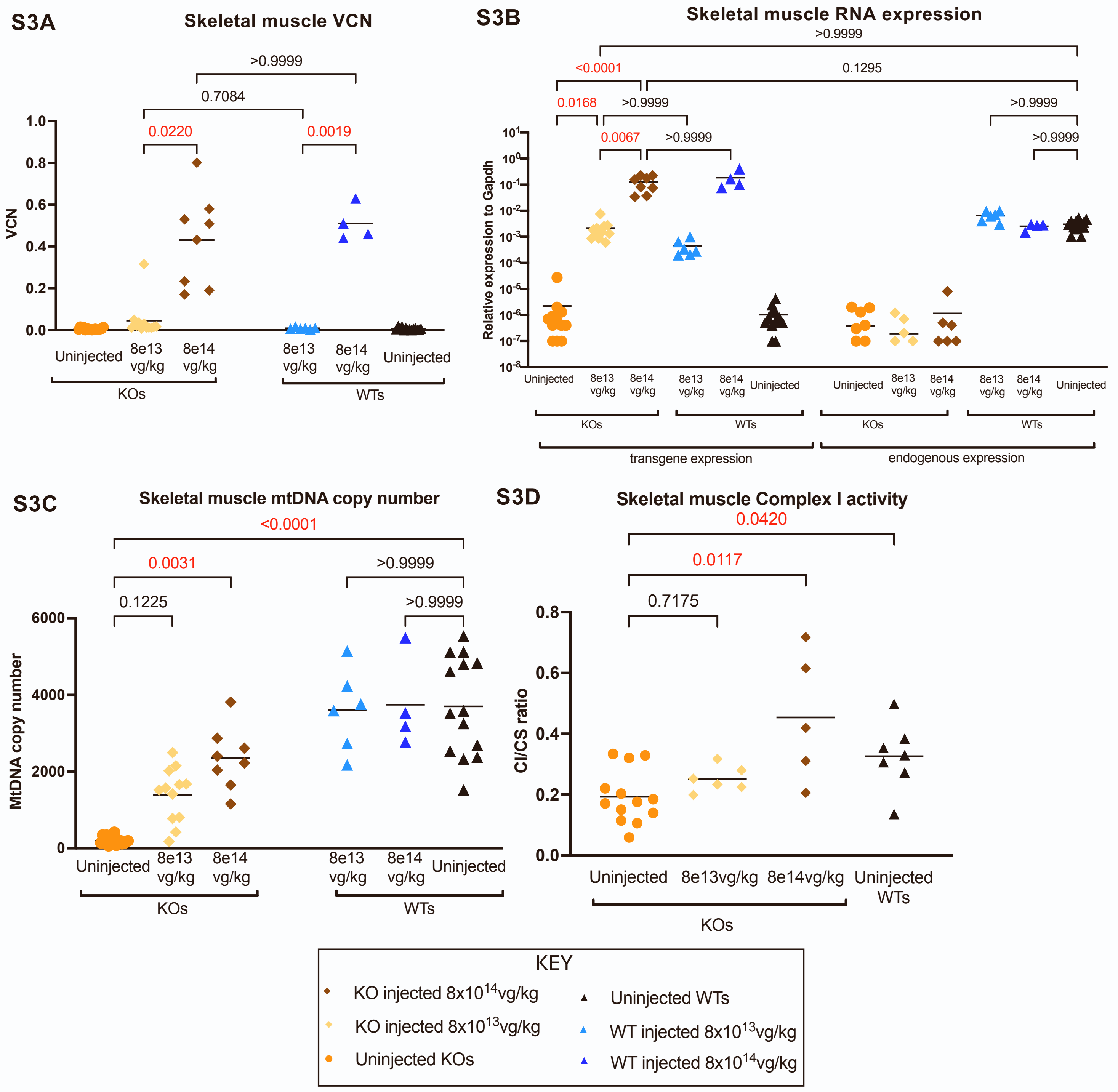

Figure S3 Outcomes of neonatal gene transfer in skeletal muscle.
S3A Skeletal muscle VCN data in injected KO and WT mice. VCN data show low but dose-dependent cell transduction in skeletal muscle following IV gene transfer. **S3B** Skeletal muscle RNA expression data. Skeletal muscle transgene (*hDGUOK)* expression in KOs injected at 8e13vg/kg and 8e14vg/kg is not statistically different to endogenous WT *mDguok* RNA expression**. S3C** Skeletal muscle mtDNA copy number data in injected KO and WT mice**.** mtDNA copy number is partially rescued following IV gene transfer at a dose of 8e14vg/kg. Sample sizes for VCN, RNA expression and mtDNA copy number studies: 16 uninjected KOs, 12 KOs injected with 8e13vg/kg, 8 KOs injected with 8e14vg/kg, 6 WT injected with 8e13vg/kg, 4 WT injected with 8e14vg/kg, 14 uninjected WTs. Statistics: Kruskal-Wallis test with multiple comparisons. **S3D** Skeletal muscle complex I activity in injected KO mice. Complex I deficiency is rescued in KOs injected at the 8e14vg/kg dose but not the 8e13vg/kg dose. Sample sizes: 13 uninjected KOs, 6 KOs injected with 8e13vg/kg, 5 KOs injected with 8e14vg/kg, 7 uninjected WTs. Statistics: Kruskal-Wallis test with multiple comparisons.

Figure S4

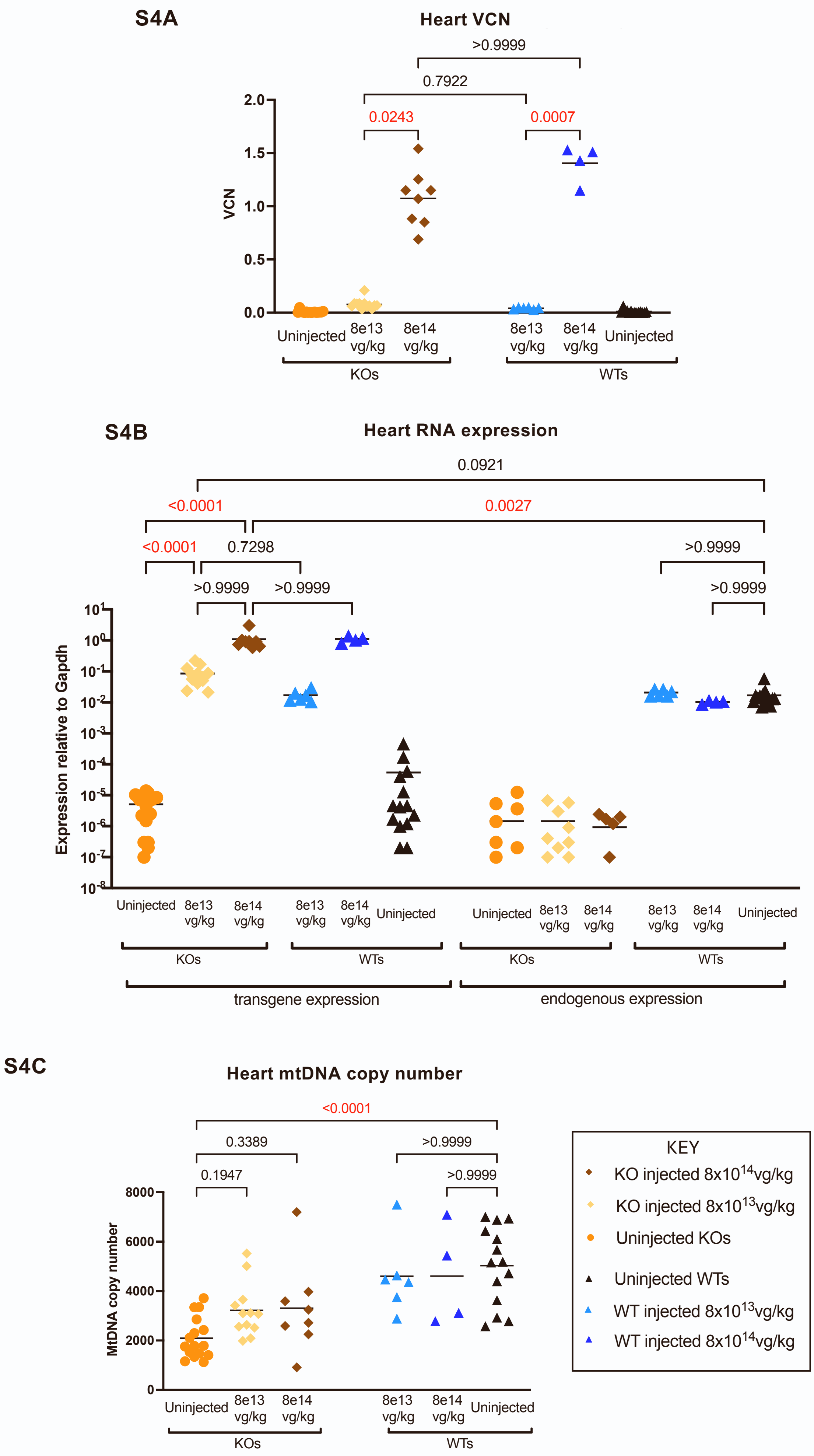

S4A
Heart VCN
>0.9999
0.7922
0.0243
0.0007
VCN
2.0
1.5
1.0
0.5
0.0
Uninjected
8e13 vg/kg
8e14 vg/kg
8e13 vg/kg
8e14 vg/kg
Uninjected
KOs
WTs
S4B
Heart RNA expression
0.0921
<0.0001
0.0027
<0.0001
0.7298
>0.9999
>0.9999
>0.9999
>0.9999
>0.9999
Expression relative to Gapdh
Uninjected
8e13 vg/kg
8e14 vg/kg
8e13 vg/kg
8e14 vg/kg
Uninjected
Uninjected
8e13 vg/kg
8e14 vg/kg
8e13 vg/kg
8e14 vg/kg
Uninjected
KOs
WTs
KOs
WTs
transgene expression
endogenous expression
S4C
Heart mtDNA copy number
<0.0001
0.3389
>0.9999
0.1947
>0.9999
MtDNA copy number
8000
6000
4000
2000
0
Uninjected
8e13 vg/kg
8e14 vg/kg
8e13 vg/kg
8e14 vg/kg
Uninjected
KOs
WTs
KEY
KO injected 8x10^14vg/kg
KO injected 8x10^13vg/kg
Uninjected KOs
Uninjected WTs
WT injected 8x10^13vg/kg
WT injected 8x10^14vg/kg

Figure S4 Outcomes of neonatal gene transfer in heart.

S4A Cardiac VCN data in injected KO and WT mice. VCN data show good transduction at doses of 8e14vg/kg following IV gene transfer.

S4B Cardiac RNA expression data in injected KO and WT mice**.** Cardiac transgene (*hDGUOK)* expression in KOs injected at 8e13vg/kg is not statistically different to endogenous WT *mDguok* RNA expression whereas KOs injected at 8e14vg/kg demonstrated higher transgene expression compared to endogenous WT *mDguok* expression**.**

S4C Cardiac mtDNA copy number data in injected KO and WT mice. mtDNA copy number is not significantly rescued following IV gene transfer.

Sample sizes for VCN, RNA expression and mtDNA copy number studies: 16 uninjected KOs, 12 KOs injected with 8e13vg/kg, 8 KOs injected with 8e14vg/kg, 6 WT injected with 8e13vg/kg, 4 WT injected with 8e14vg/kg, 14 uninjected WTs. Statistics: Kruskal-Wallis test with multiple comparisons.

Figure S5

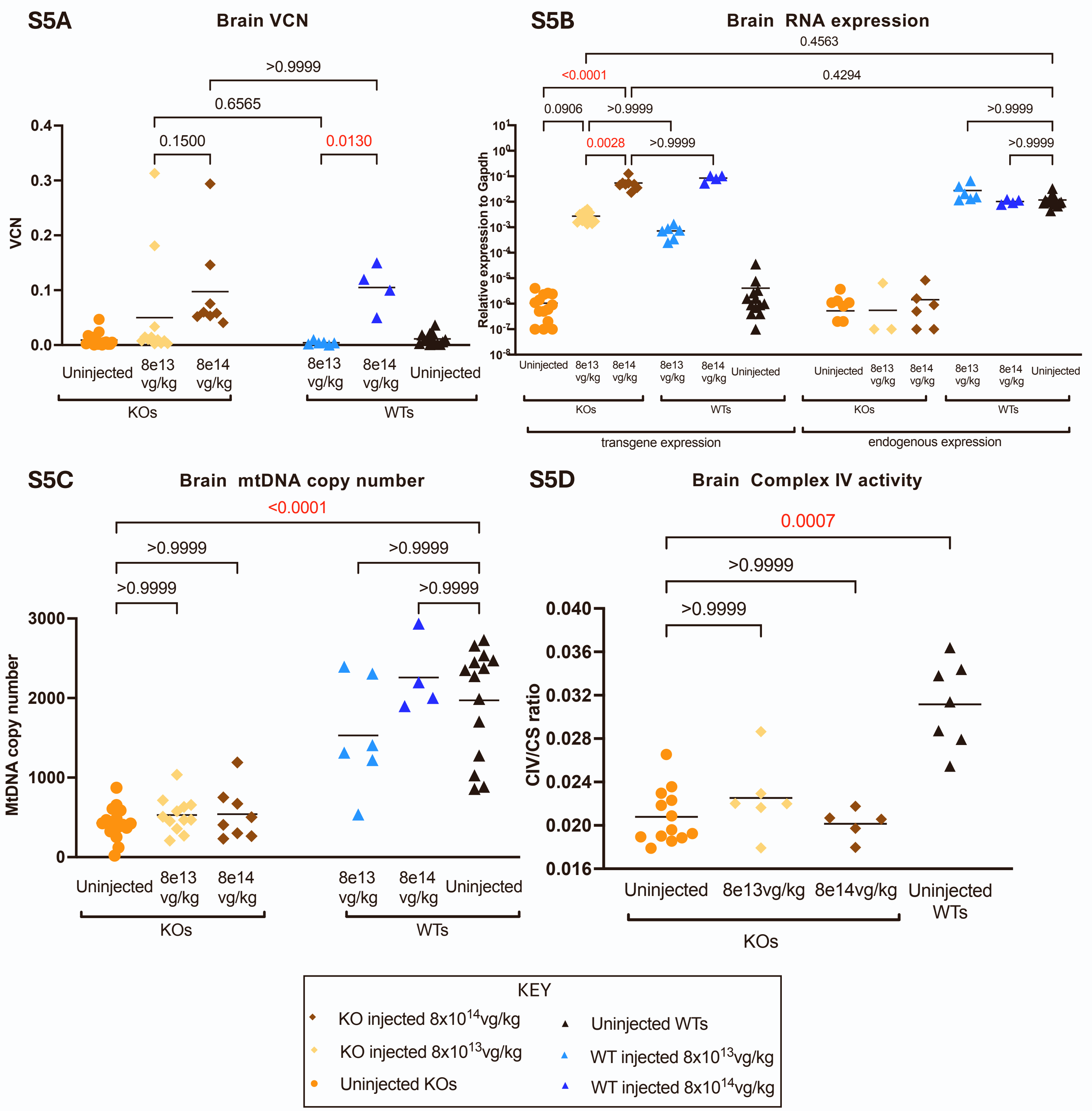
S5A
Brain VCN
VCN
>0.9999
0.6565
0.1500
0.0130
Uninjected
8e13 vg/kg
8e14 vg/kg
KOs
WTs
S5B
Brain RNA expression
Relative expression to Gapdh
0.4563
<0.0001
0.4294
0.0906
>0.9999
0.0028
transgene expression
endogenous expression
S5C
Brain mtDNA copy number
MtDNA copy number
<0.0001
S5D
Brain Complex IV activity
CIV/CS ratio
0.0007
8e13vg/kg
8e14vg/kg
Uninjected WTs
KEY
KO injected 8x10^14vg/kg
KO injected 8x10^13vg/kg
Uninjected KOs
Uninjected WTs
WT injected 8x10^13vg/kg
WT injected 8x10^14vg/kg

Figure S5 Brain outcomes following neonatal gene transfer
S5A Brain VCN data in injected KO and WT mice. VCN data show low cell transduction in brain following IV gene transfer. **S5B** Brain RNA expression data. Brain transgene (*hDGUOK)* expression in KOs injected at 8e13vg/kg and 8e14vg/kg is not statistically different to endogenous WT *mDguok* RNA expression**. S5C** Brain mtDNA copy number data in injected KO and WT mice**.** mtDNA copy number is not rescued following IV gene transfer. Sample sizes for VCN, RNA expression and mtDNA copy number studies: 16 uninjected KOs, 12 KOs injected with 8e13vg/kg, 8 KOs injected with 8e14vg/kg, 6 WTs injected with 8e13vg/kg, 4 WTs injected with 8e14vg/kg, 14 uninjected WTs. Statistics: Kruskal-Wallis test with multiple comparisons. **S5D** Brain complex IV activity in injected KO mice. Complex IV deficiency is persistent in injected KO mice despite IV gene transfer. Sample sizes: 13 uninjected KOs, 6 KOs injected with 8e13vg/kg, 5 KOs injected with 8e14vg/kg, 7 uninjected WTs. Statistics: Kruskal-Wallis test with multiple comparisons.

Table S1 OXPHOS measurements in baseline phenotyping
Data are expressed as mean±SD. Significantly decreased activity (indicated in blue) is seen in liver in KOs compared to WTs for complex I (p=0.0006), complex II+III (p=0.038), complex III (p=0.024), complex IV (p=0.002), in brain for complex IV (p=0.03) and in skeletal muscle for complex I (p= 0.0013). Significantly increased citrate synthase activity (indicated in red) is seen in liver of KOs compared to WTs, p=0.008. Sample size: 6 mice per genotype. Statistics performed using one-way ANOVA for multiple comparisons.

Organ		Complex I ratio	Complex II+III ratio	Complex III ratio	Complex IV ratio	Citrate synthase nmol/min/mg
**Liver**	KO	0.348±0.07	0.151±0.05	0.034±0.01	0.023±0.005	62±7
	HET	0.541±0.06	0.475±0.13	0.089±0.03	0.063±0.013	55±13
	WT	0.789±0.17	0.424±0.15	0.084±0.01	0.083±0.027	41±6
**Brain**	KO	0.154±0.03	0.055±0.02	0.033±0.01	0.014±0.001	170±21
	HET	0.218±0.02	0.052±0.03	0.058±0.007	0.021±0.001	180±32
	WT	0.177±0.03	0.048±0.02	0.046±0.01	0.020±0.003	179±61
**Skeletal muscle**	KO	0.185±0.05	0.080±0.04	0.020±0.02	0.014±0.003	74±15
	HET	0.324±0.07	0.144±0.02	0.034±0.02	0.019±0.006	70±24
	WT	0.423±0.08	0.140±0.04	0.045±0.02	0.019±0.004	86±31
**Heart**	KO	0.529±0.10	0.034±0.01	0.009±0.004	0.007±0.001	397±56
	HET	0.436±0.14	0.019±0.01	0.008±0.005	0.005±0.002	463±113
	WT	0.451±0.05	0.027±0.02	0.006±0.002	0.005±0.001	463±53
